# Machine learning risk stratification strategy for multiple myeloma: Insights from the EMN–HARMONY Alliance platform

**DOI:** 10.1002/hem3.70228

**Published:** 2025-10-09

**Authors:** Adrian Mosquera Orgueira, Marta Sonia Gonzalez Perez, Mattia D'Agostino, David A. Cairns, Alessandra Larocca, Juan José Lahuerta Palacios, Ruth Wester, Uta Bertsch, Anders Waage, Elena Zamagni, Carlos Pérez Míguez, Javier Alberto Rojas Martínez, Elias K. Mai, Davide Crucitti, Hans Salwender, Daniele Dall'Olio, Gastone Castellani, Manuel Piñeiro Fiel, Sara Bringhen, Sonja Zweegman, Michele Cavo, Sofía Iqbal, Jesus Maria Hernandez Rivas, Benedetto Bruno, Gordon Cook, Martin F. Kaiser, Hartmut Goldschmidt, Niels W. C. J. Van De Donk, Graham Jackson, Jesús F. San‐Miguel, Mario Boccadoro, Maria‐Victoria Mateos, Pieter Sonneveld

**Affiliations:** ^1^ Department of Hematology, IDIS University Hospital of Santiago de Compostela Santiago de Compostela Spain; ^2^ Division of Hematology, AOU Città della Salute e della Scienza di Torino University of Torino Torino Italy; ^3^ Department of Molecular Biotechnology and Health Sciences University of Torino Torino Italy; ^4^ Cancer Research UK Clinical Trials Unit, Leeds Institute of Clinical Trials Research University of Leeds Leeds United Kingdom; ^5^ Department of Molecular Biotechnology and Health Sciences, Division of Hematology University of Torino Torino Italy; ^6^ Hospital Universitario 12 de Octubre CIBER‐ONC CB16/12/00369, CNIO Madrid Spain; ^7^ Department of Hematology Erasmus MC Cancer Institute Rotterdam The Netherlands; ^8^ National Center for Tumor Diseases Heidelberg Heidelberg Germany; ^9^ Department of Medicine V, Heidelberg Myeloma Center University Hospital Heidelberg Heidelberg Germany; ^10^ Institute of Clinical and Molecular Medicine Norwegian University of Science and Technology and St. Olavs Hospital Trondheim Norway; ^11^ Department of Medical and Surgical Sciences, “Seràgnoli” Institute of Hematology University of Bologna Bologna Italy; ^12^ IRCCS Azienda Ospedaliero‐Universitaria di Bologna Bologna Italy; ^13^ Department of Internal Medicine V, Hematology, Oncology and Rheumatology, University Hospital Heidelberg GMMG Study Group Heidelberg Germany; ^14^ Asklepios Tumorzentrum Hamburg Asklepios Hospital Hamburg Altona and St. Georg Hamburg Germany; ^15^ Department of Medical and Surgical Sciences (DIMEC) University of Bologna Bologna Italy; ^16^ AOU Città della Salute e della Scienza di Torino Torino Italy; ^17^ Department of Hematology, Amsterdam UMC, Cancer Center Amsterdam Vrije Universiteit Amsterdam Amsterdam The Netherlands; ^18^ IRCCS Azienda Ospedaliero‐Universitaria di Bologna Institute of Hematology “L. e A. Seràgnoli” Bologna Italy; ^19^ Department of Medical and Surgical Sciences University of Bologna Bologna Italy; ^20^ Medical Information Scientific Engagement Johnson & Johnson Innovative Medicines New York New York United States; ^21^ Department of Medicine, Cancer Research Center (IBMCC, USAL‐CSIC), Institute of Biomedical Research of Salamanca (IBSAL) University of Salamanca Salamanca Spain; ^22^ The Institute of Cancer Research and the Royal Marsden Hospital London United Kingdom; ^23^ Department of Haematology Newcastle University Newcastle Upon Tyne United Kingdom; ^24^ Clínica Universidad de Navarra CIMA, IDISNA, CIBERONC (CB16/12/00369) Pamplona Spain; ^25^ European Myeloma Network (EMN) Italy

## Abstract

Traditional risk stratification in multiple myeloma (MM) relies on clinical and cytogenetic parameters but has limited predictive accuracy. Machine learning (ML) offers a novel approach by leveraging large datasets and complex variable interactions. This study aimed to develop and validate novel ML‐driven prognostic scores for newly diagnosed MM (NDMM), with the goal of improving upon existing ones. To this end, we analyzed data from the EMN–HARMONY MM cohort, comprising 14,345 patients, including 10,843 NDMM patients enrolled across 16 clinical trials. Three ML models were developed: (1) a comprehensive model incorporating 20 variables, (2) a reduced model including six key variables (age, hemoglobin, β2‐microglobulin, albumin, 1q gain, and 17p deletion), and (3) a cytogenetics‐free model. All models were internally validated using out‐of‐bag cross‐validation and externally validated with data from the Myeloma XI trial. Model performance was evaluated using the concordance index (C‐index) and time‐dependent area under the receiver operating characteristic curve (ROC‐AUC). The comprehensive model achieved C‐index values of 0.666 (training) and 0.667 (test) for overall survival (OS) and 0.620/0.627 for progression‐free survival (PFS). The reduced model maintained accuracy (OS: 0.658/0.657; PFS: 0.608/0.614). The cytogenetics‐free model showed C‐index values of 0.636/0.643 for OS and 0.600/0.610 for PFS. Incorporating treatment type and best response to first‐line treatment further improved performance. The new prognostic models improved over the International Staging System (ISS), Revised International Staging System (R‐ISS), and Second Revision of the International Staging System (R2‐ISS) and were reproducible in real‐world and relapsed/refractory MM, including daratumumab‐treated patients. This ML‐based risk stratification strategy provides individualized risk predictions, surpassing traditional group‐based methods and demonstrating broad applicability across patient subgroups. An online calculator is available at https://taxonomy.harmony-platform.eu/riskcalculator/.

## Graphical abstract

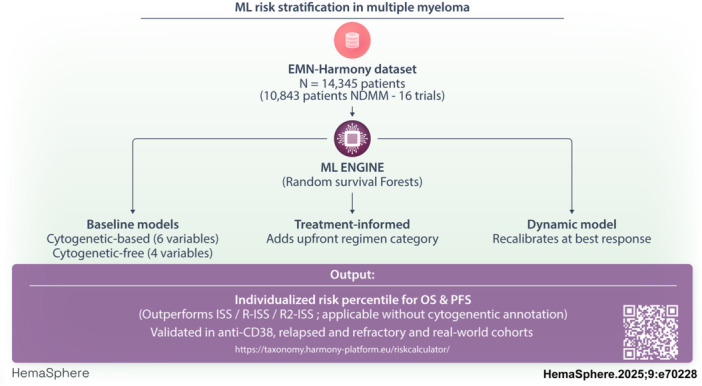

## INTRODUCTION

Multiple myeloma (MM) is a hematologic malignancy characterized by the clonal proliferation of plasma cells within the bone marrow, leading to a spectrum of clinical manifestations, including osteolytic lesions, renal dysfunction, anemia, and immunodeficiency.^1^ Despite significant advancements in therapeutic strategies—including proteasome inhibitors (PIs), immunomodulatory drugs, and monoclonal antibodies—MM remains largely incurable, with a median overall survival (OS) of approximately 5–10 years, depending on baseline prognostic factors.^2^ Although risk‐adapted treatment is not yet standard in MM, accurate risk stratification remains crucial, as it informs treatment decisions, facilitates personalized patient management, and serves as a key stratification variable in the design of new clinical trials.^3^

Traditional MM risk stratification models, such as International Staging System (ISS),^4^ International Myeloma Working Group (IMWG),^5^ Revised International Staging System (R‐ISS),^6^ or the newer Mayo Additive Staging System (MASS)^7^ and Second Revision of the International Staging System (R2‐ISS),^8^ stratify patients into broad risk groups based on a limited set of clinical and cytogenetic variables. However, these models lack granularity, as they do not provide individualized, patient‐specific risk estimates or fully exploit the richness of available clinical and cytogenetic data. Additionally, they fail to incorporate treatment‐specific variables, despite their known influence on prognosis, and remain static, without dynamic recalibration as disease status evolves.^9^, ^10^, ^11^ These limitations underscore the need for more refined, data‐driven approaches to MM risk stratification.

Machine learning (ML) presents a transformative opportunity for risk stratification in MM by leveraging large‐scale datasets and identifying complex, nonlinear relationships among multiple prognostic variables.^12^ ML algorithms can process high‐dimensional data, refine prognostic accuracy, and generate individualized risk assessments that surpass traditional models.^13^ While ML‐driven prognostic models have demonstrated potential in oncology,^14^, ^15^, ^16^ their implementation in MM remains limited due to challenges in data heterogeneity, model interpretability, and the need for robust validation across diverse patient populations. The HARMONY Consortium,^17^ integrating data from multiple clinical trials and patient registries, offers an unprecedented platform to address these challenges. By integrating clinical and cytogenetic data with longitudinal follow‐up and treatment response information, this initiative enables the development of ML‐based prognostic tools with improved generalizability and clinical applicability.

This study introduces an ML strategy for risk stratification in newly diagnosed multiple myeloma (NDMM), trained on the large multicenter dataset from the EMN–HARMONY Consortium. Relying exclusively on data routinely collected in standard care, the model outperforms established scoring systems and delivers more accurate prognostic estimates for patients treated across diverse clinical settings. Importantly, it requires no molecular inputs and maintains high predictive accuracy even when cytogenetic data are unavailable, maximizing its practicality for everyday use.

## MATERIALS AND METHODS

### Data source and sample size justification

This study used data from the EMN–HARMONY Consortium, a collaborative initiative under the European Myeloma Network that integrates clinical and cytogenetic information from multiple sources. Prior to data access, the research team submitted a predefined analysis proposal—detailing objectives, modeling strategy, and evaluation plan—to the EMN–HARMONY Scientific Committee. The proposal was reviewed and approved, ensuring methodological transparency and minimizing the risk of selective reporting.

The analyzed dataset comprised 14,345 MM patients, comprising 10,843 NDMM patients from 16 clinical trials and 3502 patients from relapsed/refractory trials and real‐world registries (Supporting Information S1: Data 1). To ensure direct comparability, the dataset was partitioned into training and test sets following the same methodology as that used for the development of the R2‐ISS score,^8^ with the Myeloma XI,^18^ VISTA,^19^ and POLLUX^20^ trials serving as the external validation cohorts. In addition, further evaluations were performed on 2221 real‐world patients from the EMN–HARMONY dataset (Table 1).

**Table 1:** Summary of clinical trials included in the EMN–HARMONY cohort, detailing autologous stem cell transplantation (ASCT) eligibility and inclusion in the previous Second Revision of the International Staging System (R2‐ISS) study.^8^

Study	*N*	Part of the R2‐IIS study	ASCT eligibility
*EMN01*	654	Yes	No
*EMN02/HO95 MM*	1493	Yes	Yes
*GEM05MAS65*	259	Yes	No
*GEM05MENO65*	389	Yes	Yes
*GEM2010MAS65*	236	Yes	No
*GIMEMA‐MM‐03‐05*	511	Yes	No
*HOVON 65 MM*	826	Yes	Yes
*HOVON 87 MM*	630	Yes	No
*IST‐CAR‐506*	58	Yes	No
*MM‐BO2005*	474	Yes	Yes
*MM5*	502	Yes	Yes
*MMY2069*	152	Yes	No
*Myeloma XI*	3771	Yes	Both
*RV‐MM‐EMN‐441*	387	Yes	Yes
*RV‐MM‐PI‐114*	102	Yes	Yes
*RV‐MM‐PI‐209*	399	Yes	Yes
Wilhelminen Hospital	24	No	‐
Triple Class Refractory			
La Fe Hospital	84	No	‐
Triple Class Refractory			
Med University Hannover	19	No	‐
Triple Class Refractory			
Registry of Gammapathies in Castilla and León	2094	No	‐
*VISTA*	682	No	‐
*POLLUX*	599	No	‐
Total	14,345	10,843	‐

Written informed consent was obtained from all patients before enrollment in the source trials, which were approved by the institutional review boards and ethics committees of all participating centers and conducted in accordance with the Declaration of Helsinki. After the acquisition of data from the source trials, all patient data were de facto anonymized in compliance with the General Data Protection Regulation, harmonized and transformed using an Observational Medical Outcomes Partnership Common Data Model, and subsequently integrated in the EMN–HARMONY platform.

Twenty predictors common to all studies in the database were used. According to the quantitative framework by Riley et al. for time‐to‐event prediction models,^21^ a 20‐predictor survival model with anticipated *R*² = 0.20 and 35% cumulative mortality at 6.6 years requires approximately 5000 participants (~1700 deaths) to ensure a global shrinkage ≥0.90, discrimination optimism ≤0.01, and ≤10% relative risk variance. Our development cohort supplies 7702 patients, with 3214 deaths and 5332 progression events, yielding ~161 and 267 events per predictor for OS and progression‐free survival (PFS), respectively—well above both the classical ≥10 and the ≥50 events‐per‐predictor thresholds recommended for ML models—and an expected shrinkage of ≈0.98.

### Development of the baseline risk stratification tools

**Figure 1:**
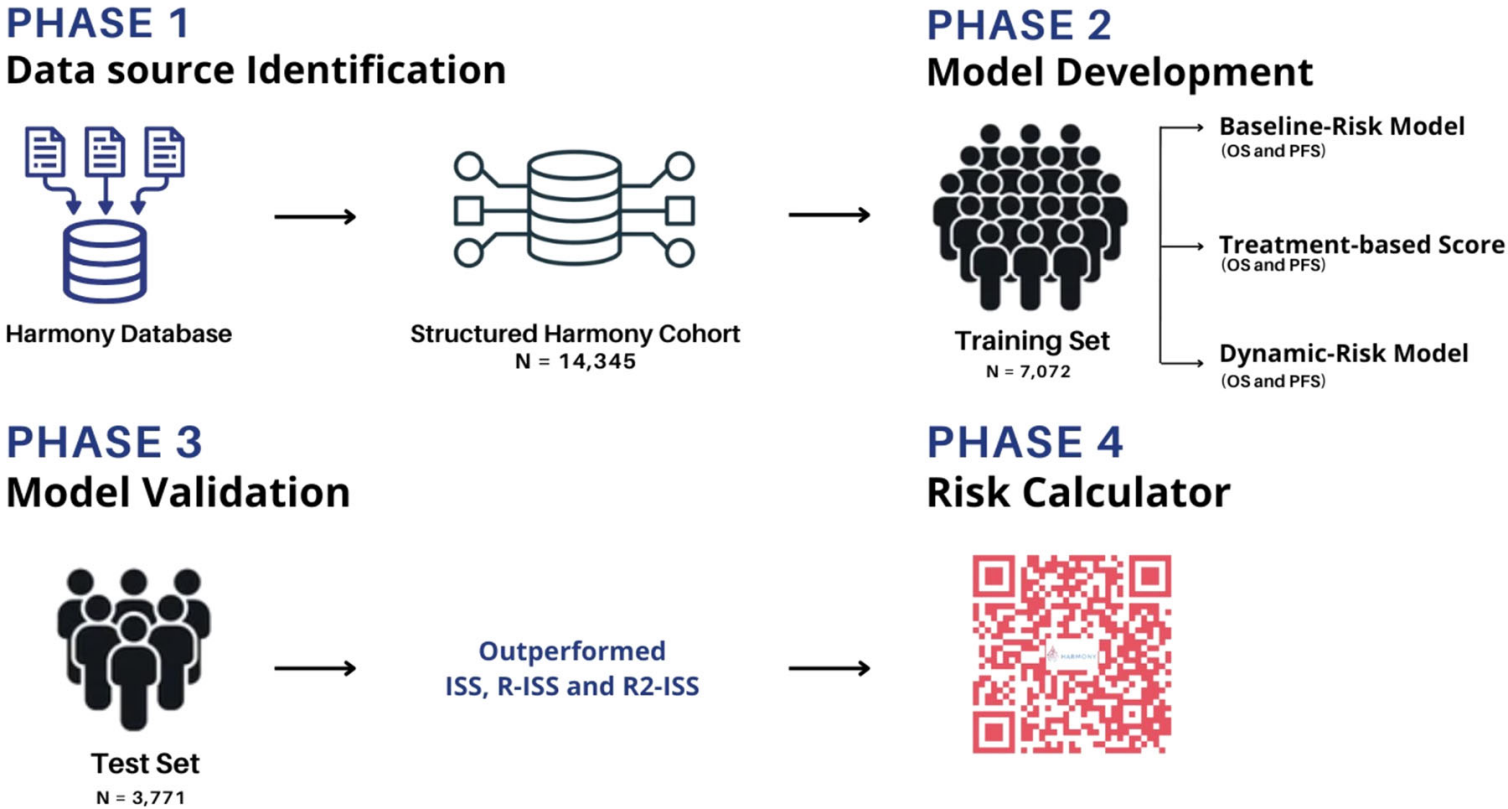
**Graphical workflow of the study, outlining the methodology used for data preprocessing, model development, validation, and performance evaluation.** ISS, International Staging System; OS, overall survival; PFS, progression‐free survival; R‐ISS, Revised International Staging System; R2‐ISS, Second Revision of the International Staging System.

The risk stratification tool was developed by integrating a comprehensive set of clinical, cytogenetic, and biochemical variables to capture the complex prognostic landscape of MM (Figure 1). Clinical variables included patient age, hemoglobin, beta‐2 microglobulin, albumin, and upfront treatment data. Cytogenetic markers encompassed key recurrent abnormalities including 1q gain and 17p deletion. Biochemical parameters included lactate dehydrogenase (LDH) levels and renal function markers. To address missing data, a Random Forest model^22^ was used for baseline variable imputation, preserving critical prognostic information and enhancing data completeness (see Supporting Information S1: Data 2 for details on the computational environment and software versions).

To construct the risk stratification models, a Random Survival Forest (RSF) algorithm^23^ was used for its ability to handle high‐dimensional data and model complex, nonlinear interactions among variables. The EMN–Harmony Consortium dataset was used to train the RSF model, with variable importance metrics guiding the selection of predictive features. Hyperparameter tuning (number of variables randomly sampled at each split and minimum terminal node size) was performed using a standard grid‐search procedure, selecting the combination that maximized the out‐of‐bag (OOB) concordance index (C‐index). Dimensionality reduction was performed to eliminate redundant or weakly informative variables, thereby enhancing the model's efficiency and interpretability. The final model was optimized to maximize discriminative ability while minimizing the number of variables, ensuring high predictive accuracy and clinical feasibility.

### Validation of the baseline risk stratification tools

The developed risk stratification tools were subjected to rigorous validation to ensure their robustness and generalizability across diverse patient populations (Figure 1). Internal validation aimed at preventing overfitting was performed using OOB cross‐validation within the training set, leveraging the bootstrapping mechanism of the RSF algorithm to obtain unbiased performance estimates. External validation was conducted using data from the Myeloma XI,^18^ VISTA,^19^ and POLLUX^20^ trials, which served as independent validation cohorts. Furthermore, an independent real‐world cohort of 2221 patients from the EMN–HARMONY dataset (Table 1) was used for external validation, with analyses restricted to OS due to the lack of PFS annotation. These external validations were essential for assessing the model's generalizability and ensuring consistent predictive accuracy across diverse clinical settings and patient populations.

Model discrimination was quantified using the C‐index and the time‐dependent area under the receiver‐operating‐characteristic curve (time‐dependent AUC). The C‐index measures how well predicted risks order observed survival times, ranging from 0.5 (no discrimination) to 1.0 (perfect discrimination); 95% confidence intervals (CIs) were derived with the *concordance* function of the survival package in R.^24^, ^25^ The time‐dependent AUC evaluates discrimination at successive time points, providing a dynamic assessment of predictive accuracy, and was computed using the timeROC package.^26^ Both metrics were computed for OS and PFS prediction models.

To account for events that preclude the primary outcome, we fit Fine–Gray subdistribution hazard models, defining disease progression as the event of interest and death prior to progression as the competing event. Cumulative‐incidence functions, along with subdistribution‐specific C‐index and time‐dependent AUC values, were estimated using the riskRegression package in R.^27^, ^28^, ^29^

Model calibration was assessed by ranking individual‐level predicted risks and grouping them into deciles of equal size. Within each decile, the mean predicted event probability (“expected”) was compared visually with the observed event rates estimated from Kaplan–Meier curves at the target horizon. Calibration was evaluated graphically without formal estimation of the calibration intercept or slope.

### Visualization of risk score

To visualize the distribution of risk scores, we first computed the 25th (Q1), 50th (Q2), and 75th (Q3) percentiles of the OOB risk estimates in the training cohort for both OS and PFS, separately for the cytogenetics‐based and cytogenetics‐free models. Patients were then grouped into quartiles (Q1–Q4) based on these thresholds, which were applied unchanged to the independent Myeloma XI test set. Kaplan–Meier curves for each end point were plotted by quartile to depict survival across the four risk strata.

### Comparison with existing models

To evaluate the performance of the ML‐based risk stratification model against established prognostic scoring systems, we compared C‐indexes and time‐dependent AUCs. The analysis was restricted to patients with complete annotations for ISS, R‐ISS, and R2‐ISS variables to minimize potential bias from imputation. This approach ensured a fair comparison, accurately reflecting the predictive capabilities of each model and highlighting the added prognostic value of the ML‐driven prognostic scores.

### Development of treatment‐informed and dynamic risk scores

To further refine prognostic accuracy, we developed a treatment‐informed risk score incorporating upfront therapy regimens, followed by a dynamic risk score integrating response to first‐line therapy (Figure 1). The treatment‐informed score was constructed by analyzing patients treated with PIs, immunomodulatory imides (IMiDs), or PI–IMiD combinations. These treatment categories were used to recalibrate the baseline risk models, thereby generating treatment‐specific prognostic scores. This approach accounts for the impact of initial therapy on outcomes, potentially improving the model's predictive performance. Owing to the adaptive design of the Myeloma XI trial, patients could not be unambiguously assigned to a single first‐line treatment category; therefore, treatment‐specific results for this cohort are not presented.

For the dynamic model, we included only patients with a documented best response to first‐line therapy. PFS and OS times were re‐anchored at the date of that response, classified as complete response (CR) or better, very good partial response (VGPR), partial response (PR), stable disease (SD), or progressive disease (PD). Landmarking survival in this way mitigates immortal‐time bias and provides a conditional risk estimate applicable to the clinical scenario in which a response has been confirmed. Patients who progressed before an evaluable response or lacked response data were not included in this analysis, as they fall outside the model's intended scope. Risk estimation and recalibration were carried out using the RSF algorithm. Best response data were not available for the Myeloma XI cohort within the EMN–HARMONY dataset; consequently, dynamic model results for that trial are not reported.

### Implementation and deployment of the web‐based risk calculator data

The risk calculator was developed as a web‐based interactive application using Shiny,^30^ featuring a modular UI‐server architecture to ensure scalability, flexibility, and seamless integration with the ML prognostic models. The workflow follows a structured sequence where users first select the desired risk model—baseline, treatment‐informed, or dynamic. Patient‐specific variables are then entered and validated against predefined clinical ranges. The selected RSF model computes an individual risk score that is immediately translated into a percentile rank relative to the full training cohort. This percentile reflects *relative* risk: for example, a value at the 80th percentile indicates that the patient's risk score is higher than 80% of the training population. For added clinical context, the percentile is also displayed separately for the transplant‐eligible and transplant‐ineligible subgroups of that same cohort. Results are visualized as color‐coded horizontal bars, enabling rapid, intuitive interpretation of patient‐specific risk stratification for PFS and OS.

## RESULTS

### Study population characteristics

This study included 14,345 patients with MM from the EMN–HARMONY Consortium, comprising 10,843 newly diagnosed patients from 16 clinical trials, 2221 patients from real‐world registries, and 1281 patients in trials for relapsed or refractory MM (Table 1, Supporting Information S1: Data 1). Of these, 7702 newly diagnosed patients comprised the training set, including 4572 (64.6%) classified as transplant‐eligible and 2500 (35.3%) as transplant‐ineligible. The test set was derived from the Myeloma XI^18^ trial and included 3771 patients, encompassing both transplant‐eligible and transplant‐ineligible individuals.

In the training set, 35.8% of patients were classified as ISS stage I (low risk), 39.6% as stage II (intermediate risk), and 24.6% as stage III (high risk). By comparison, in the test set, 25.7% of patients were categorized as ISS stage I, 42.2% as stage II, and 32.1% as stage III. According to the R‐ISS system, 13.5%, 68.8%, and 17.7% of patients in the training set were classified as low‐, intermediate‐, and high‐risk categories, respectively, whereas in the test set, these proportions were 12.9%, 71.2%, and 15.8%, respectively. The median age was 62 years (interquartile range [IQR] 14.2 years) in the training set and 68 years (IQR 14.0 years) in the test set.

Over a median follow‐up of 6.6 years (95% CI, 6.5–6.7) in the training set, the median OS was 7.2 years (95% CI, 7.0–7.5) and the median PFS was 2.8 years (95% CI, 2.7–2.9). In the test set, the median follow‐up was 5.4 years (95% CI, 5.3–5.6), with a median OS of 5.6 years (95% CI, 5.4–5.9) and a median PFS of 2.0 years (95% CI, 2.0–2.1).

When the entire set of variables was considered, the overall missing data rate was 35.4% in the training set and 45.6% in the test set, with substantial variation across variable types (Table 2). To address these gaps, Random Forest–based imputation was performed, ensuring inclusion of all patients in subsequent analyses and reducing potential bias from missing data.

**Table 2:** Overview of missing data across the variables analyzed in the EMN–HARMONY cohort, specifying the proportion of unavailable data for each predictor.

Variable	Non missing	Missing	Missing data (%)
Age	12,956	1389	9.68
1q gain	4330	10,015	69.82
1p del	1351	12,994	90.58
13q del	4975	9370	65.32
17p del	7203	7142	49.79
t(11;14)	4853	9492	66.17
t(14;16)	5875	8470	59.04
t(4;14)	7039	7306	50.93
t(6;14)	5873	8472	59.06
Hemoglobin	3284	11,061	77.11
Leukocytes	3256	11,089	77.3
Platelets	3256	11,089	77.3
Albumin	13,842	503	3.51
LDH	11,020	3325	23.18
Beta‐2 microglobulin	12,292	2053	14.31
Monoclonal spike	1903	12,442	86.73
Involved free‐light chain	1341	13,004	90.65
Urine monoclonal spike	1146	13,199	92.01
Bone marrow plasma cells	7320	7025	48.97
ISS	11,986	2359	16.44

Abbreviations: ISS, International Staging System; LDH, lactate dehydrogenase.

### Development and performance of the baseline risk models

Initially, we developed a comprehensive RSF model using the full set of variables, incorporating 20 clinical, cytogenetic, and biochemical variables to estimate OS and PFS (Table 3). The full model demonstrated clinically relevant discrimination, achieving a C‐index of 0.666 (95% CI, 0.656–0.675) for OS in the training set and 0.667 (95% CI, 0.651–0.677) in the test set. For PFS, the model achieved a C‐index of 0.620 (95% CI, 0.612–0.628) in the training set and 0.627 (95% CI, 0.613–0.635) in the test set.

**Table 3:** Concordance index (C‐index) values for overall survival and progression‐free survival predictions using the baseline and dynamic risk models developed in the study.

Model	End point	Training set (C‐index)	Test set (C‐index)
Complete model (20 variables)	OS	0.666 (0.656–0.675)	0.667 (0.651–0.677)
	PFS	0.620 (0.612–0.628)	0.627 (0.613–0.635)
Reduced model (6 variables)	OS	0.658 (0.646–0.666)	0.657 (0.642–0.668)
	PFS	0.608 (0.604–0.620)	0.614 (0.600–0.622)
Cytogenetics‐free model (12 variables)	OS	0.645 (0.635–0.655)	0.654 (0.641–0.668)
	PFS	0.604 (0.596–0.612)	0.624 (0.613–0.635)
Cytogenetics‐free model (4 variables)	OS	0.636 (0.629–0.649)	0.643 (0.642–0.668)
	PFS	0.600 (0.592–0.608)	0.61 (0.600–0.622)
Treatment‐oriented risk model (cytogenetics‐based)	OS	0.664 (0.653–0.674)	—
	PFS	0.623 (0.614–0.631)	—
Treatment‐oriented risk model (cytogenetics‐free)	OS	0.645 (0.634–0.655)	—
	PFS	0.607 (0.598–0.616)	—
Dynamic risk model (cytogenetics‐based)	OS	0.699 (0.689–0.709)	—
	PFS	0.701 (0.693–0.708)	—
Dynamic risk model (cytogenetics‐free)	OS	0.682 (0.672–0.692)	—
	PFS	0.693 (0.684–0.699)	—

Abbreviations: OS, overall survival; PFS, progression‐free survival.

**Figure 2:**
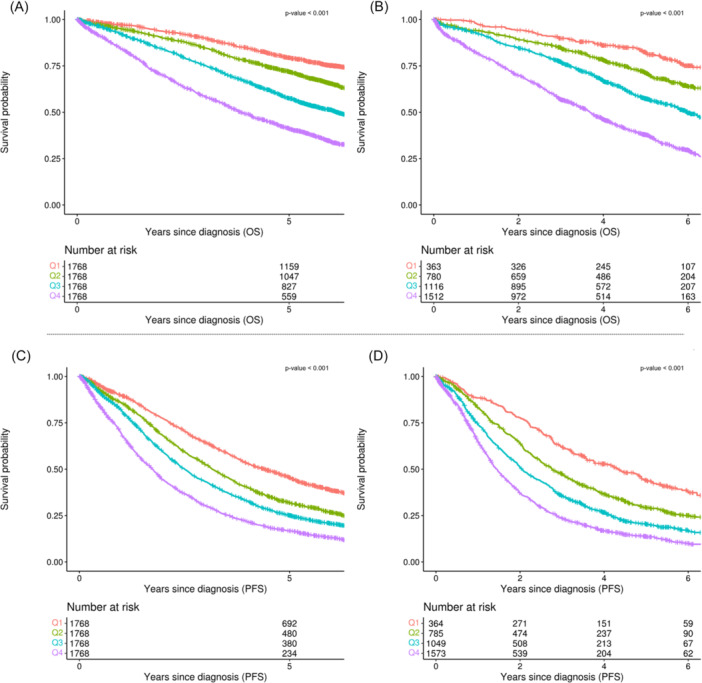
**Kaplan–Meier curves for the cytogenetics‐based model, with patients stratified into quartiles (Q1–Q4, determined by the 25th, 50th, and 75th percentiles) of continuous risk scores calculated separately for overall survival (OS) and progression‐free survival (PFS) prediction. (A)** OS in the training cohort, **(B)** OS in the test cohort, **(C)** PFS in the training cohort, and **(D)** PFS in the test cohort.

**Figure 3:**
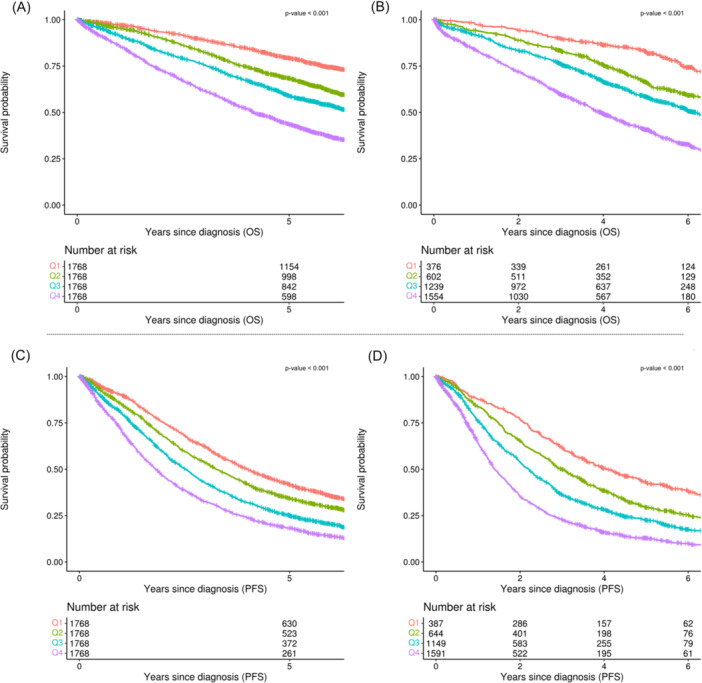
**Kaplan–Meier curves for the cytogenetics‐free model, with patients stratified into quartiles (Q1–Q4, determined by the 25th, 50th, and 75th percentiles) of continuous risk scores calculated separately for overall survival (OS) and progression‐free survival (PFS) prediction. (A)** OS in the training cohort, **(B)** OS in the test cohort, **(C)** PFS in the training cohort, and **(D)** PFS in the test cohort.

To refine the model while maintaining predictive accuracy, we used variable importance measures derived from the RSF algorithm to reduce the number of predictors. The resulting reduced model retained six key predictors (age, hemoglobin, beta‐2 microglobulin, albumin, 1q gain, and 17p deletion), achieving a C‐index of 0.658 (95% CI, 0.646–0.666) for OS in the training set and 0.657 (95% CI, 0.642–0.668) in the test set, and for PFS, it achieved 0.608 (95% CI, 0.604–0.620) and 0.614 (95% CI, 0.600–0.622), respectively (Figures 2 and 3; Supporting Information S2: Figure 1 and Supporting Information S3: Figure 2).

Additionally, we explored a cytogenetics‐free model by excluding all cytogenetic variables during training. This 12‐variable model maintained strong predictive performance, with a C‐index of 0.645 (95% CI, 0.635–0.655) for OS in the training set and 0.654 (95% CI, 0.641–0.668) in the test set, and 0.604 (95% CI, 0.596–0.612) and 0.624 (95% CI, 0.613–0.635) for PFS, respectively. Further dimensionality reduction produced a four‐variable model—retaining the same non‐cytogenetic predictors (age, hemoglobin, beta‐2 microglobulin, and albumin) as the cytogenetics‐based model—achieving a C‐index of 0.636 (95% CI, 0.629–0.649) for OS in the training set and 0.643 (95% CI, 0.642–0.668) in the test set, and 0.600 (95% CI, 0.592–0.608) and 0.610 (95% CI, 0.600–0.622) for PFS, respectively (Figures 2 and 3).

A subsequent grid search over the number of variables randomly sampled at each split and terminal‐node size showed that hyperparameter tuning marginally improved the C‐index by <0.01 in all scenarios—differences entirely within the default model's 95% CIs—so the default RSF settings were retained.

### Sensitivity analysis for missing‐data burden

To assess the robustness of our models with respect to the extent of missing data subject to imputation, we stratified patients according to their proportion of missing values (>50% vs. ≤50%) for both the cytogenetics‐based and cytogenetics‐free models. In the cytogenetics‐based model, 1.6% of training patients and 20.2% of test patients had >50% missing data; C‐indexes were lower (OS: 0.60 vs. 0.66 in training; 0.63 vs. 0.66 in test; PFS: 0.57 vs. 0.62 in training; 0.58 vs. 0.61 in test), yet they continued to demonstrate discrimination. In the cytogenetics‐free model, the high‐missingness subgroup comprised 0.9% of the training cohort and 0.1% of the test cohort; discrimination declined (OS: C‐index 0.55 vs. 0.64 in training; PFS: 0.60 vs. 0.61) but remained prognostic, although the test subset was small. Complete C‐index estimates with 95% CIs are provided in Supporting Information S9: Table 1. These results indicate that, although model performance attenuates as the degree of missingness increases, substantial prognostic information is preserved.

### Performance of the baseline risk stratification models

**Figure 4:**
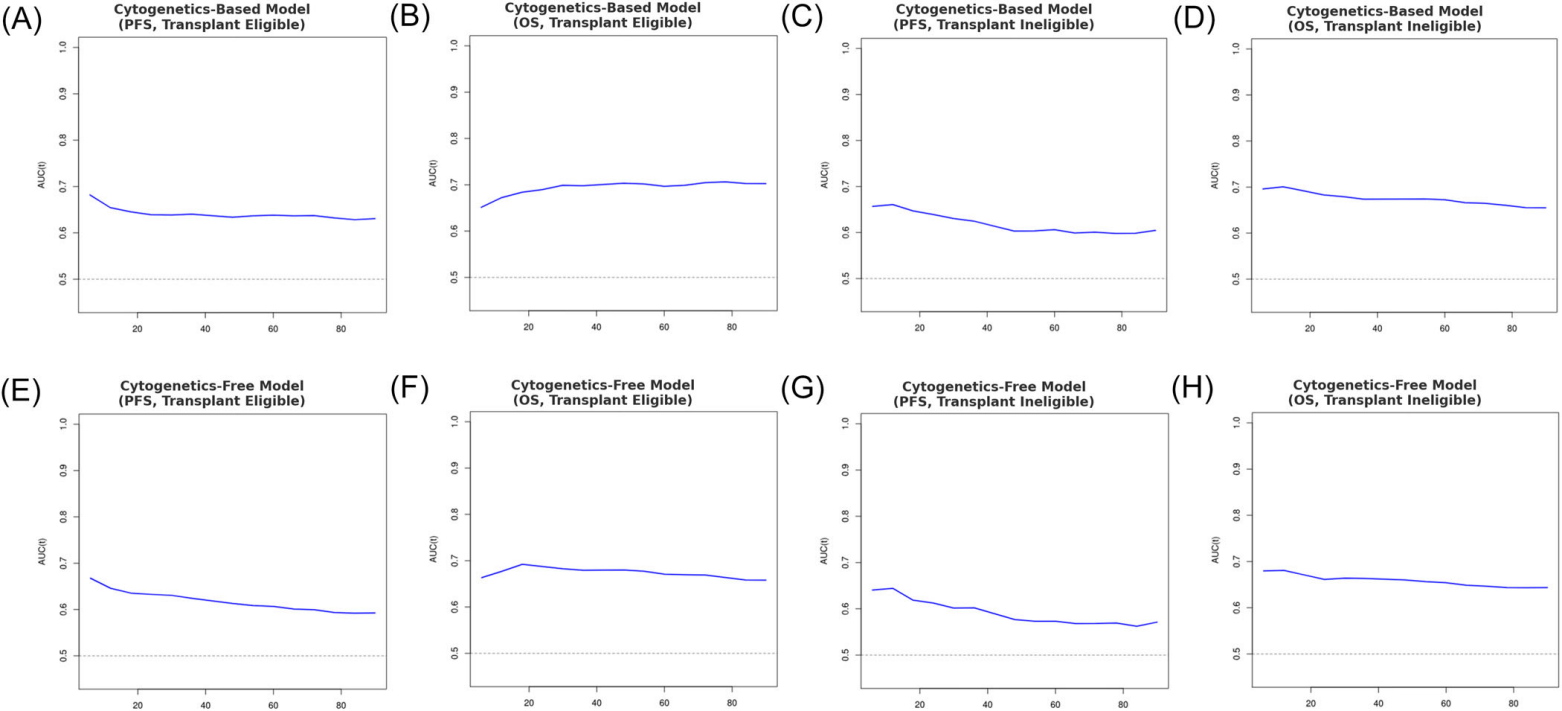
Time‐dependent area under the receiver‐operating‐characteristic curves (AUCs) evaluating the accuracy of the baseline cytogenetics‐based and cytogenetics‐free models for overall survival (OS) and progression‐free survival (PFS) prediction in transplant‐eligible and transplant‐ineligible patients.

Both the cytogenetics‐based and cytogenetics‐free prognostic models showed superior predictive performance for OS and PFS compared to the ISS, R‐ISS, and R2‐ISS scores (Table 4, Supporting Information S4: Figure 3, Supporting Information S5: Figure 4, and Supporting Information S10: Table 2). The cytogenetics‐based model achieved higher C‐indexes and time‐dependent AUCs for both end points, indicating improved discriminative ability. Similarly, the cytogenetics‐free model also outperformed traditional staging systems, maintaining strong predictive accuracy even without cytogenetic variables. Importantly, both models showed similar accuracy across transplant‐eligible and transplant‐ineligible patient subsets, supporting their robustness and applicability across clinical settings (Figure 4). Notably, all comparisons with conventional scores were restricted to patients with complete data for the respective scoring systems, thereby avoiding potential biases from imputation and ensuring that the observed performance differences reflect genuine improvements in prognostic accuracy.

**Table 4:** Comparative analysis of the baseline risk models (cytogenetics‐based and cytogenetics‐free) against the International Staging System (ISS), Revised International Staging System (R‐ISS), and Second Revision of the International Staging System (R2‐ISS) scores in both the training and test sets.

Model	Conventional score training set	ML score training set	Δ	Conventional score test set	ML score test set	Δ
*(A) Cytogenetics‐based model*						
ML versus ISS (OS)	0.609 (0.599–0.619)	0.655 (0.646–0.665)	0.05	0.608 (0.594–0.621)	0.656 (0.643–0.670)	0.05
ML versus ISS (PFS)	0.576 (0.568–0.583)	0.609 (0.600–0.617)	0.03	0.577 (0.566–0.588)	0.609 (0.598–0.620)	0.03
ML versus R‐ISS (OS)	0.625 (0.613–0.636)	0.664 (0.651–0.677)	0.05	0.597 (0.579–0.615)	0.654 (0.634–0.674)	0.06
ML versus R‐ISS (PFS)	0.587 (0.577–0.597)	0.615 (0.604–0.626)	0.03	0.563 (0.548–0.578)	0.612 (0.596–0.629)	0.05
ML versus R2‐ISS (OS)	0.647 (0.630–0.663)	0.661 (0.643–0.678)	0.01	0.614 (0.592–0.636)	0.674 (0.652–0.696)	0.06
ML versus R2‐ISS (PFS)	0.608 (0.595–0.622)	0.618 (0.604–0.632)	0.01	0.567 (0.549–0.585)	0.617 (0.598–0.635)	0.05
*(B) Cytogenetics‐free model*						
ML versus ISS (OS)	0.609 (0.599–0.619)	0.638 (0.627–0.648)	0.03	0.608 (0.594–0.621)	0.642 (0.628–0.656)	0.03
ML versus ISS (PFS)	0.576 (0.568–0.583)	0.594 (0.585–0.602)	0.02	0.577 (0.566–0.588)	0.607 (0.596–0.619)	0.03
ML versus R‐ISS (OS)	0.625 (0.613–0.636)	0.640 (0.626–0.653)	0.02	0.597 (0.579–0.615)	0.624 (0.603–0.645)	0.03
ML versus R‐ISS (PFS)	0.587 (0.577–0.597)	0.593 (0.582–0.604)	0.01	0.563 (0.548–0.578)	0.602 (0.585–0.618)	0.04
ML versus R2‐ISS (OS)	0.647 (0.630–0.663)	0.641 (0.623–0.658)	−0.04	0.614 (0.592–0.636)	0.639 (0.615–0.662)	0.03
ML versus R2‐ISS (PFS)	0.608 (0.595–0.622)	0.601 (0.586–0.615)	−0.01	0.567 (0.549–0.585)	0.611 (0.593–0.630)	0.04

*Note*: Comparisons are based on concordance index (C‐index) values within a subset of patients with complete data for the classical models.

Abbreviations: ML, machine learning; OS, overall survival; PFS, progression‐free survival.

To further assess model reproducibility, we assessed their performance in the VISTA^19^ and POLLUX^20^ trials, which included patients with relapsed or refractory MM. Importantly, POLLUX included participants treated with daratumumab‐based therapy, providing an additional benchmark in a modern treatment context. Both models maintained their prognostic accuracy despite differences in disease biology and prior treatment exposure, supporting their validity in this patient population (Table 5). Notably, in the POLLUX cohort, both the cytogenetics‐based and cytogenetics‐free models maintained strong discrimination in the DRd arm (OS C‐index 0.653 [95% CI, 0.601–0.705] and 0.625 [95% CI, 0.570–0.680], respectively), whereas the Rd control arm showed slightly higher C‐index values (OS C‐index 0.686 [95% CI, 0.640–0.732] and 0.675 [95% CI, 0.627–0.722]). Nevertheless, predictive accuracy in the DRd arm remained robust for both OS and PFS, underscoring the models' relevance in contemporary daratumumab‐based therapeutic settings. These findings reinforce the generalizability of the ML‐based risk stratification scores across different disease stages and treatment regimens.

**Table 5:** Performance of the cytogenetics‐based and cytogenetics‐free models in predicting overall survival and progression‐free survival in relapsed or refractory multiple myeloma (MM) patients from the VISTA and POLLUX trials, measured by concordance index (C‐index) values.

		VISTA			POLLUX		
Model	End point	Global	VMP arm	MP arm	Global	DRd arm	Rd arm
Cytogenetics‐based model (6 variables)	OS	0.640 (0.589–0.691)	0.598 (0.512–0.684)	0.669 (0.606–0.732)	0.669 (0.634–0.704)	0.653 (0.601–0.705)	0.686 (0.640–0.732)
	PFS	0.639 (0.594–0.683)	0.639 (0.567–0.710)	0.637 (0.578–0.696)	0.629 (0.598–0.660)	0.636 (0.590–0.682)	0.640 (0.596–0.684)
Cytogenetics‐free model (4 variables)	OS	0.629 (0.576–0.683)	0.595 (0.510–0.681)	0.655 (0.587–0.724)	0.652 (0.616–0.689)	0.625 (0.570–0.680)	0.675 (0.627–0.722)
	PFS	0.646 (0.601–0.690)	0.653 (0.583–0.722)	0.647 (0.588–0.705)	0.608 (0.576–0.641)	0.597 (0.548–0.647)	0.626 (0.581–0.671)

Abbreviations: DRd, daratumumab + lenalidomide + dexamethasone; MP, melphalan + prednisone; OS, overall survival; PFS, progression‐free survival; Rd, lenalidomide + dexamethasone; VMP, bortezomib + melphalan + prednisone.

External validation in an unselected real‐world cohort of 2221 patients confirmed the models' performance in predicting OS: the cytogenetics‐based model achieved a C‐index of 0.675 (95% CI, 0.659–0.690) and the cytogenetics‐free model achieved a C‐index of 0.673 (95% CI, 0.657–0.689), which were close to the values observed in the clinical‐trial datasets, thereby supporting their external validity.

### Variable importance in cytogenetics‐based and cytogenetics‐free baseline models

**Figure 5:**
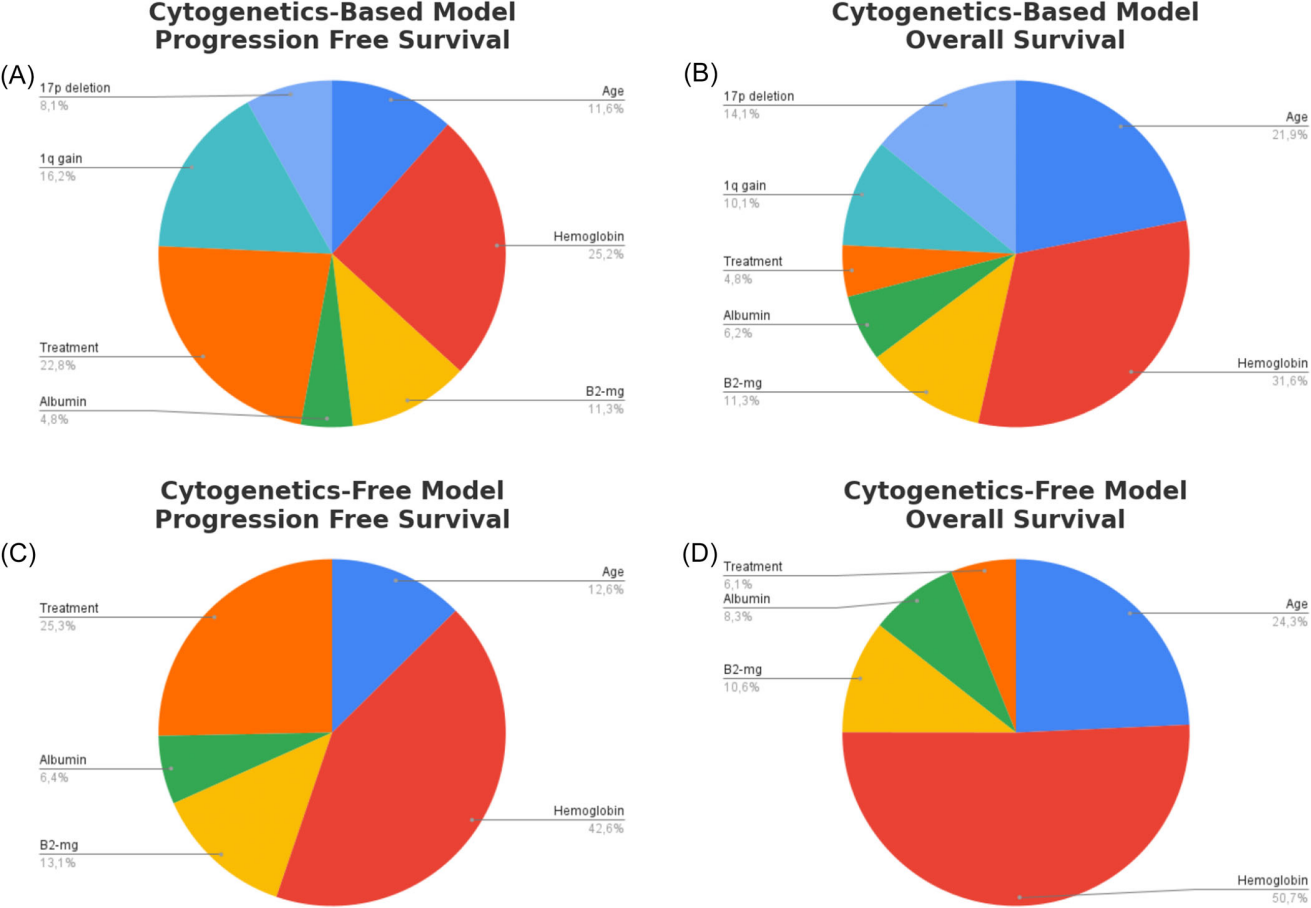
**Variable‐importance analysis based on the permutation metric implemented in the *vimp* function of the *randomForestSRC* R package.** Bars represent the drop in out‐of‐bag prediction accuracy observed after permuting each predictor, thereby quantifying its relative contribution to model performance. Results are shown for overall survival and progression‐free survival predictions generated by the cytogenetics‐based and cytogenetics‐free Random Survival Forest models.

In both the cytogenetics‐based and cytogenetics‐free models, hemoglobin, age, and beta‐2 microglobulin emerged as the most influential predictors of OS and PFS, highlighting their fundamental prognostic role in MM (Table 6, Figure 5). While the cytogenetics‐based model incorporated 1q gain and 17p deletion, their relative contribution to the model's discriminative ability was lower than that of the primary clinical predictors. Notably, the absence of cytogenetic markers did not substantially alter the hierarchy of predictive variables, as hemoglobin, age, and beta‐2 microglobulin remained the top contributors within the cytogenetics‐free model. These findings underscore the robustness of clinical and biochemical factors in MM prognostication, demonstrating their predictive strength even in the absence of cytogenetic data.

**Table 6:** Variable‐importance analysis showing the contribution of each predictor in the cytogenetics‐based and cytogenetics‐free baseline models.

Variable	OS (%)	PFS (%)
*Cytogenetics‐based model*		
Age	29.02	24.84
Hemoglobin	20.52	18.31
Beta‐2 microglobulin	17.88	20.67
17p deletion	12.54	8.49
1q gain	10.51	16.35
Albumin	9.54	11.33
*Cytogenetics‐free model*		
Age	31.27	30.55
Hemoglobin	21.36	21.57
Beta‐2 microglobulin	25.88	29.89
Albumin	21.49	17.99

Abbreviations: OS, overall survival; PFS, progression‐free survival.

### Impact of incorporating upfront treatment on prognostic accuracy

The impact of upfront treatment variables on PFS and OS prediction was evaluated in the training set, given the complexity and response‐adapted design of the Myeloma XI^18^ trial (Table 3). Among 5823 patients, treatment regimens were unambiguously categorized as IMiD‐based (*N* = 3078), PI‐based (*N* = 1139), or PI‐IMiD‐based (*N* = 1606). In the cytogenetics‐based model, the OOB C‐index improved modestly from 0.660 (95% CI, 0.649–0.670) to 0.665 (95% CI, 0.655–0.675) for OS prediction and from 0.608 (95% CI, 0.600–0.617) to 0.628 (95% CI, 0.619–0.636) for PFS prediction with the addition of treatment data (Supporting Information S6: Figure 5). Similarly, in the cytogenetics‐free model, incorporating treatment variables marginally increased the OOB C‐index from 0.642 (95% CI, 0.631–0.652) to 0.646 (95% CI, 0.636–0.657) for OS, and from 0.593 (95% CI, 0.584–0.602) to 0.611 (95% CI, 0.602–0.620) for PFS. These findings suggest that while upfront treatment contributes moderately to PFS prognostication, the RSF models retain strong predictive accuracy using only baseline clinical and cytogenetic variables, supporting their applicability even in the absence of detailed treatment data.

### Incorporating treatment response into dynamic risk prediction models

**Figure 6:**
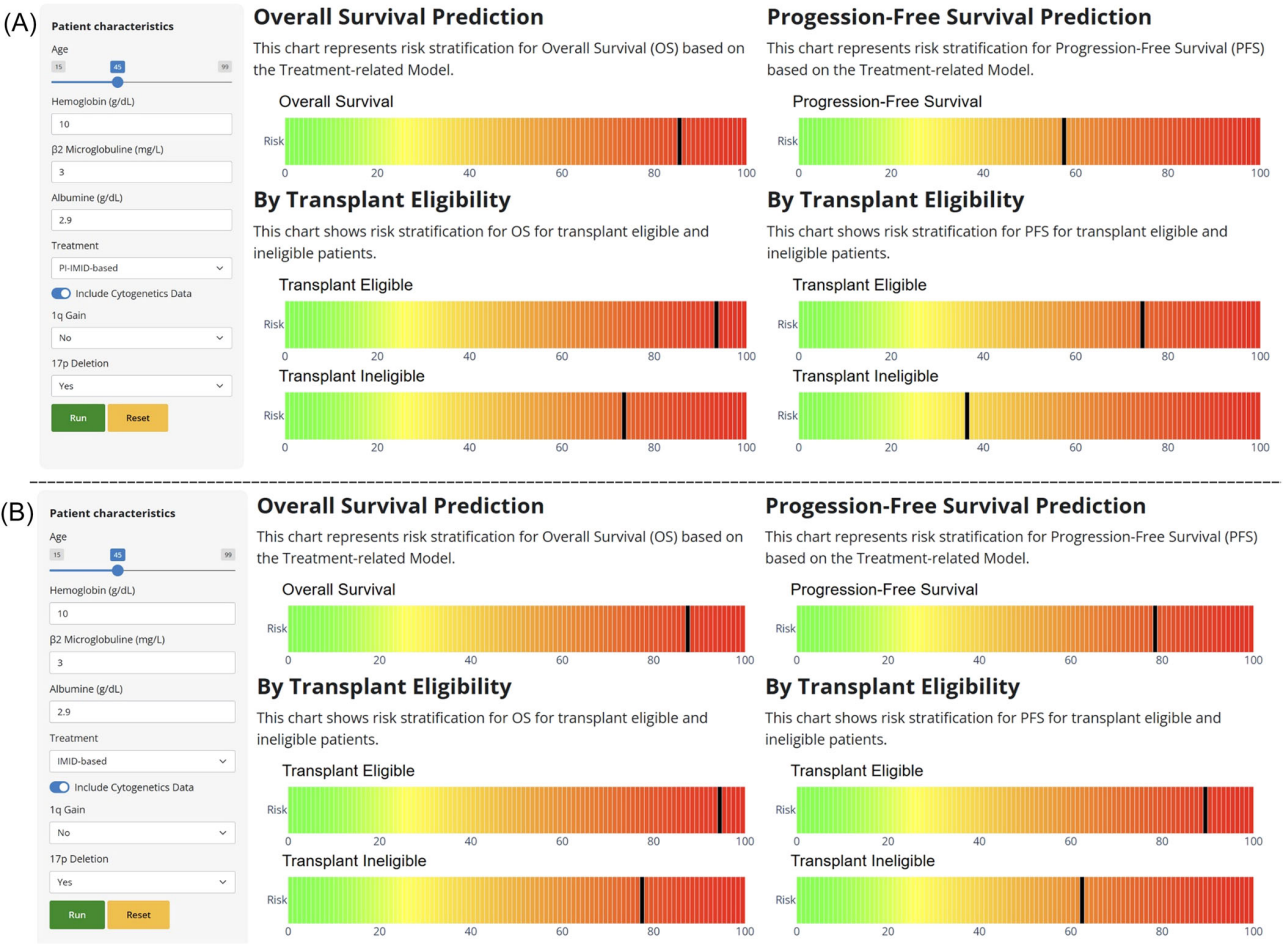
**Example output of the risk score calculator for a patient with hemoglobin (Hb) 10 g/dL, beta‐2 microglobulin (B2‐mg) 3.0 mg/dL, albumin 2.9 mg/dL, presence of 17p deletion, and absence of 1q gain. (A, B)** The predicted risk distributions for immunomodulatory imide (IMiD)‐based therapy (A) and proteasome inhibitor (PI)–IMiD combination therapy (B). For each regimen, the patient's Random Forest–derived risk score is converted into a percentile rank relative to the corresponding distribution in the training cohort. Percentiles are displayed overall and stratified by transplant eligibility status in the training set (transplant candidate vs. noncandidate), providing an intuitive frame of reference for clinicians. Lower percentiles indicate lower relative risk (e.g., for progression‐free survival), whereas higher percentiles indicate higher relative risk. Because the metric is percentile‐based, it represents relative—not absolute—risk.

Recalibrating risk predictions from the time of best treatment response significantly improved prognostic accuracy in both OS and PFS (Table 3). Among 6518 patients with documented best response, integrating these data into the cytogenetics‐based model increased the C‐index from 0.654 to 0.698 (95% CI, 0.688–0.708) for OS and from 0.604 to 0.702 (95% CI, 0.695–0.710) for PFS (Supporting Information S7: Figure 6). Similar improvements were observed in the cytogenetics‐free model, where the C‐index for OS increased from 0.636 (95% CI, 0.625–0.646) to 0.683 (95% CI, 0.673–0.693) and for PFS from 0.591 (95% CI, 0.583–0.600) to 0.695 (95% CI, 0.687–0.702). These findings underscore the strong prognostic value of integrating treatment response data derived from serological parameters, revealing that dynamic risk modeling substantially improves predictive accuracy, even in the absence of cytogenetic information.

### Deployment of the EMN‐HARMONY Myeloma Risk Calculator

To evaluate the clinical utility of the calculator, all six ML‐based models (baseline, treatment‐informed, and dynamic, each available in cytogenetics‐based and cytogenetics‐free versions) were deployed within an online platform (Figure 6, Supporting Information S8: Figure 7). The platform allows users to enter patient‐specific parameters and obtain OS and PFS estimates under different treatment scenarios. By incorporating both baseline risk factors and dynamic response data, the tool provides nuanced, individualized prognostic estimates. Examples of its application illustrated how treatment selection and response to induction therapy can influence risk stratification, offering valuable insights for clinical decision‐making.

## DISCUSSION

In this study, we developed and validated a data‐driven, ML‐based risk stratification strategy for MM, integrating cytogenetics‐based and cytogenetics‐free models to improve prognostic accuracy for OS and PFS. These models outperformed traditional risk scores (ISS,^4^ R‐ISS,^6^ and R2‐ISS^8^) by leveraging a broader array of clinical, biochemical, and cytogenetic variables. Hemoglobin, beta‐2 microglobulin, albumin, and age emerged as the most influential factors, in line with state‐of‐the‐art literature,^4^, ^31^, ^32^, ^33^ while cytogenetic markers such as 1q gain and 17p deletion further enhanced risk prediction, consistent with previous reports.^8^, ^34^, ^35^, ^36^ Importantly, the models demonstrated consistent performance across transplant‐eligible and transplant‐ineligible patients, underscoring their versatility. Incorporating treatment‐related variables provided additional prognostic refinement, particularly for PFS, reinforcing the relevance of upfront therapy in risk assessment. Moreover, the dynamic risk model, recalibrated at the time of best response to first‐line therapy, significantly enhanced prognostication. Unlike conventional staging systems, which rely solely on static variables, this strategy incorporates baseline, treatment‐informed, and dynamic models, offering a more adaptable and personalized approach to risk stratification.^9^, ^11^, ^37^

Beyond NDMM, we also validated our baseline models in real‐world cohorts and in relapsed or refractory MM patients, including those treated in the VISTA and POLLUX trials.^19^, ^38^ The models maintained strong predictive accuracy in this population, even among patients receiving daratumumab‐based regimens, highlighting their applicability across different disease stages and treatment settings. These findings underscore the generalizability of this ML‐driven risk stratification strategy, supporting its integration into clinical workflows to support patient management, evidence‐based treatment decisions, and clinical trial design in MM.

Recently, genomic signatures such as the IMS‐Barcelona classifier^39^ and the IrMMa signature^40^ have shown additional prognostic value in MM. Incorporating such genomic information into ML‐based risk tools could further enhance predictive accuracy when those data are available. However, a major advantage of the proposed strategy lies in its capacity to provide accurate risk estimates without the need for genomic sequencing or FISH, thereby enhancing its applicability in resource‐limited settings and clinical environments with limited access to advanced diagnostic technologies.

Another key advantage of this strategy lies in its implementation as a web‐based risk calculator, serving as a digital biomarker that delivers real‐time, evidence‐based risk assessments to support clinical decision‐making. Unlike static scoring systems, this complementary interactive tool dynamically generates continuous risk estimates—rather than assigning patients to rigid categories—based on patient‐specific variables, including cytogenetic data and treatment response, thereby adding a novel dimension to risk assessment. By integrating ML‐derived risk scores into an intuitive platform, the calculator enables granular, personalized risk evaluation that evolves with the patient's disease course, offering actionable insights for treatment selection and monitoring. Its online accessibility further broadens its utility, particularly in resource‐limited settings where advanced diagnostics are often unavailable. This approach bridges the gap between conventional categorical systems and precision medicine, providing clinicians with both familiar reference points and sophisticated, adaptive risk modeling.

This study benefits from the comprehensive and diverse dataset provided by the EMN–HARMONY Consortium, integrating clinical trial and limited real‐world data from a large cohort of MM patients, thereby enhancing the reliability and generalizability of our risk stratification models. The use of ML enabled the integration of a broad range of clinical, cytogenetic, and biochemical variables, thereby improving predictive accuracy and model robustness. The availability of both cytogenetics‐based and cytogenetics‐free models further increases their applicability across different clinical settings. This approach aligns with contemporary efforts to integrate digital tools into clinical workflows, facilitating evidence‐based treatment decisions and improving patient management in clinical settings where cytogenetics or genomic data are unavailable.^41^ However, as with any retrospective analysis, selection bias and missing data remain potential limitations. While the dataset is highly representative of the MM population, external validation beyond clinical trial populations is a major concern,^42^, ^43^, ^44^, ^45^, ^46^, ^47^ and additional validation in real‐world clinical settings would further strengthen its applicability across diverse patient groups. Future studies should focus on expanding the validation of these models across broader cohorts, ensuring its continued evolution as a precise and adaptable risk stratification tool.

A key methodological consideration in this study is the management of missing data, which is inherent to large, multisource datasets such as EMN–HARMONY. Although several variables had high proportions of missingness, the absolute number of patients with available values remained consistently high, exceeding 1000 real cases for each variable. This allowed imputations to be based on sufficiently large, representative subsets, thereby enhancing their reliability. We used Random Forest–based imputation, a state‐of‐the‐art technique capable of capturing nonlinear relationships and interactions among variables without assuming parametric distributions. Notably, in the final reduced model, the variable with the highest missingness (hemoglobin) still had over 3200 complete observations. Moreover, to prevent imputation from introducing bias in model comparisons, all benchmarking against conventional prognostic scores was restricted to patients with complete data for the respective scoring systems. Importantly, even in the presence of partial information, ML models can leverage meaningful patterns from observed data—including informative missingness—without requiring perfectly imputed variables.^48^ These safeguards ensure that the predictive advantage of the ML‐based models reflects true signal rather than artifacts arising from data handling.

Future research should further refine dynamic risk modeling by incorporating minimal residual disease (MRD)^49^, ^50^, ^51^, ^52^, ^53^ assessments at multiple time points, further enhancing prognostic precision and adapting risk prediction to disease evolution. Incorporation of genetic mutations,^34^, ^54^ gene expression profiles,^55^, ^56^ circulating plasma cell counts,^57^, ^58^ and extramedullary disease detected by PET‐CT^59^, ^60^, ^61^ could also strengthen risk stratification by capturing deeper biological complexity. Additionally, although the model has already been validated in anti‐CD38‐containing regimens for relapsed/refractory disease, forthcoming first‐line datasets will enable regimen‐specific recalibration for each emerging quadruplet backbone (e.g., daratumumab‐VRd, isatuximab‐KRd), ensuring that prognostic performance remains optimized as new frontline therapies become standard of care.^62^, ^63^

## CONCLUSION

We have developed and validated an ML‐based risk stratification strategy for NDMM that surpasses ISS, R‐ISS, and R2‐ISS in predictive accuracy. Integrating a dynamic risk score based on treatment response further improved prognostic precision. To facilitate clinical adoption, we provide an interactive online calculator (https://taxonomy.harmony-platform.eu/riskcalculator/) that delivers real‐time, individualized risk assessments. Future prospective studies are warranted to validate its integration into clinical practice and confirm its impact on patient management.

## AUTHOR CONTRIBUTIONS

**Adrian Mosquera Orgueira**: Conceptualization; methodology; software; formal analysis; writing—original draft; project administration. **Marta Sonia Gonzalez Perez**: Writing—original draft; supervision; writing—review and editing. **Mattia D'Agostino**: Supervision; project administration. **David A. Cairns**: Data curation; writing—review and editing. **Alessandra Larocca**: Data curation; writing—review and editing. **Juan José Lahuerta Palacios**: Data curation; writing—review and editing. **Ruth Wester**: Writing—review and editing. **Uta Bertsch**: Data curation; writing—review and editing. **Anders Waage**: Data curation; writing—review and editing. **Elena Zamagni**: Data curation; writing—review and editing. **Carlos Pérez Míguez**: Data curation; writing—review and editing; software; formal analysis. **Javier Alberto Rojas Martínez**: Formal analysis; writing—original draft. **Elias K. Mai**: Writing—review and editing; data curation. **Davide Crucitti**: Software; data curation; formal analysis. **Hans Salwender**: Writing—review and editing; data curation. **Daniele Dall'Olio**: Data curation; writing—review and editing. **Gastone Castellani**: Data curation; writing—review and editing. **Manuel Piñeiro Fiel**: Data curation; writing—review and editing. **Sara Bringhen**: Data curation; writing—review and editing. **Sonja Zweegman**: Data curation; writing—review and editing. **Michele Cavo**: Data curation; writing—review and editing. **Sofía Iqbal**: Writing—review and editing. **Jesus Maria Hernandez Rivas**: Data curation; writing—review and editing. **Benedetto Bruno**: Data curation; writing—review and editing. **Gordon Cook**: Data curation; writing—review and editing. **Martin F. Kaiser**: Data curation; writing—review and editing. **Hartmut Goldschmidt**: Data curation; writing—review and editing. **Niels W. C. J. Van De Donk**: Data curation; writing—review and editing. **Graham Jackson**: Data curation; writing—review and editing. **Jesús F. San‐Miguel**: Data curation; supervision; writing—review and editing. **Mario Boccadoro**: Data curation; supervision; writing—review and editing. **Maria‐Victoria Mateos**: Data curation; supervision; writing—review and editing. **Pieter Sonneveld**: Data curation; supervision; writing—review and editing.

## CONFLICT OF INTEREST STATEMENT

Adrian Mosquera Orgueira: Roche: consultancy; Pfizer: consultancy; AbbVie: membership on an entity's board of directors or advisory committees, speakers bureau; AstraZeneca: consultancy, membership on an entity's board of directors or advisory committees, speakers bureau; Janssen: consultancy, membership on an entity's board of directors or advisory committees, speakers bureau; Takeda: speakers bureau; Biodigital THX: current equity holder in private company; Novartis: other; Incyte: other; GSK: consultancy. Mattia D'Agostino: GlaxoSmithKline: honoraria, membership on an entity's board of directors or advisory committees; Sanofi: honoraria, membership on an entity's board of directors or advisory committees; Bristol Myers Squibb: membership on an entity's board of directors or advisory committees; Janssen: honoraria, research funding; Adaptive Biotechnologies: membership on an entity's board of directors or advisory committees. Alessandra Larocca: Janssen: honoraria, other: participation in advisory board; GSK: honoraria, other: participation in advisory board; Menarini: honoraria, other: participation in advisory board; Sanofi: honoraria. Ruth Wester: Sanofi: honoraria; Janssen: honoraria. Hans Salwender: Janssen: honoraria, other: travel grant; Oncopeptides: honoraria; Pfizer: honoraria; Sanofi: honoraria, other: travel grant; Stemline: honoraria; Roche: honoraria; Takeda: honoraria; Chugai: honoraria; GlaxoSmithKline: honoraria; Bristol Myers Squibb/Celgene: honoraria, other: travel grant; Amgen: honoraria, other: travel grant; AbbVie: honoraria; Sebia: honoraria. Sara Bringhen: AbbVie, Amgen, Bristol Myers Squibb, GlaxoSmithKline, Janssen, and Sanofi: speakers bureau; Sanofi: consultancy, honoraria; Bristol Myers Squibb, Janssen, Oncopeptides, Pfizer, Stemline Therapeutics, and Takeda: other: participation in advisory boards. Sonja Zweegman: Sanofi: membership on an entity's board of directors or advisory committees; BMS: membership on an entity's board of directors or advisory committees; GSK: membership on an entity's board of directors or advisory committees; Janssen: membership on an entity's board of directors or advisory committees, research funding; Amgen: membership on an entity's board of directors or advisory committees; Takeda: research funding; Oncopeptides: membership on an entity's board of directors or advisory committees. Jesus Maria Hernandez Rivas: GlaxoSmithKline: consultancy, honoraria; Amgen: honoraria, membership on an entity's board of directors or advisory committees, speakers bureau; Pfizer: honoraria, membership on an entity's board of directors or advisory committees; Bristol Myers Squibb: honoraria, membership on an entity's board of directors or advisory committees, research funding, speakers bureau. Gordon Cook: Celgene: research funding; Amgen: consultancy, speakers bureau; Janssen: consultancy, research funding; Bristol Myers Squibb: consultancy, honoraria; Janssen‐Cilag: honoraria, speakers bureau; Takeda: consultancy, honoraria, research funding, speakers bureau. Martin F. Kaiser: Pfizer: consultancy, honoraria; GSK: consultancy; Sanofi: consultancy; BMS/Celgene: consultancy, honoraria, research funding; Pfizer: consultancy, honoraria; J&J/Janssen: consultancy, honoraria, research funding; Roche: consultancy; Poolbeg: consultancy, honoraria; Regeneron: consultancy. Hartmut Goldschmidt: Sanofi: honoraria, membership on an entity's board of directors or advisory committees, other: grants and/or provision of investigational medicinal product; support for attending meetings and/or travel, research funding; Chugai: honoraria, other: grants and/or provision of investigational medicinal product; Adaptive Biotechnologies: membership on an entity's board of directors or advisory committees; Millennium Pharmaceuticals Inc.: research funding; Takeda: research funding; Amgen: honoraria, membership on an entity's board of directors or advisory committees, other: support for attending meetings and/or travel; grants and/or provision of investigational medicinal product, research funding; Celgene: research funding; Bristol Myers Squibb: honoraria, membership on an entity's board of directors or advisory committees, other: support for attending meetings and/or travel, research funding; Janssen: honoraria, membership on an entity's board of directors or advisory committees, other: grants and/or provision of investigational medicinal product; support for attending meetings and/or travel, research funding; Karyopharm: research funding; Hoffmann‐La Roche: research funding; Molecular Partners: research funding; Novartis: honoraria, other: support for attending meetings and/or travel, research funding; MorphoSys AG: research funding; Bristol Myers Squibb/Celgene: other: grants and/or provision of investigational medicinal product; Merck Sharp and Dohme (MSD): research funding; GlycoMimetics Inc.: research funding; Incyte Corporation: research funding; Dietmar Hopp Foundation: other: grants and/or provision of investigational medicinal product; Array Biopharma/Pfizer: other: grants and/or provision of investigational medicinal product; GlaxoSmithKline (GSK): honoraria, other: support for attending meetings and/or travel, research funding; Heidelberg Pharma: research funding; Pfizer: honoraria, other: support for attending meetings and/or travel, research funding; Johns Hopkins University: other: grants and/or provision of investigational medicinal product; Mundipharma GmbH: other: grants and/or provision of investigational medicinal product. Pieter Sonneveld: Oncopeptides: patents and royalties; Karyopharm: membership on an entity's board of directors or advisory committees, patents and royalties, research funding; Pfizer: membership on an entity's board of directors or advisory committees, patents and royalties; European Myeloma Network: other: president; Celgene: membership on an entity's board of directors or advisory committees, research funding; Janssen: membership on an entity's board of directors or advisory committees, patents & royalties, research funding; Bristol Myers Squibb: membership on an entity's board of directors or advisory committees, research funding; Amgen: membership on an entity's board of directors or advisory committees, research funding. Jesús F. San‐Miguel: Bristol Myers Squibb: other: advisory board; Celgene: other: advisory board; Roche: other: advisory board; Janssen‐Cilag: other: advisory board; Novartis: other; Karyopharm: other: advisory board; GlaxoSmithKline: other: advisory board; Haemalogix: other: advisory board; MSD: other: advisory board; Amgen: consultancy, other: advisory board; Takeda: other: advisory board; Sanofi: other: advisory board; AbbVie: consultancy, other: advisory board; Regeneron: other: advisory board; SecuraBio: other: advisory board. Mario Boccadoro: GlaxoSmithKline: membership on an entity's board of directors or advisory committees; AbbVie: honoraria; Bristol Myers Squibb: honoraria, research funding; Janssen: honoraria, membership on an entity's board of directors or advisory committees, research funding; Novartis: honoraria, research funding; Amgen: honoraria, research funding; Celgene: honoraria, research funding; Sanofi: honoraria, research funding; Mundipharma: research funding. Maria‐Victoria Mateos: BMS/Celgene, Janssen‐Cilag, Sanofi, AbbVie, Stemline, Oncopeptides, GSK: honoraria, membership on an entity's board of directors or advisory committees; Amgen, Takeda, Regeneron: honoraria.

## ETHICS STATEMENT

Before participating in the source studies, every patient provided their written informed consent. These studies were approved by the institutional review boards (IRBs) and ethics committees at each location and were carried out in line with the Declaration of Helsinki's ethical principles. Once the data from these studies were collected, all patient information was anonymized to meet General Data Protection Regulation (GDPR) standards.

## FUNDING

This work was supported by the HARMONY and HARMONY PLUS projects (IMI2 Joint Undertaking under grant agreements No. 116026 and No. 945406), which receive funding from the European Union's Horizon 2020 Research and Innovation Programme and the European Federation of Pharmaceutical Industries and Associations (EFPIA).

## Supporting information

Supporting Information.

Supporting Information.

Supporting Information.

Supporting Information.

Supporting Information.

Supporting Information.

Supporting Information.

Supporting Information.

Supporting Information.

Supporting Information.

## Data Availability

Data collected for this analysis and related documents are available upon reasonably justified request, which needs to be written and addressed to the attention of Dr Adrian Mosquera Orgueira at the following e‐mail address: adrian.mosquera.orgueira@sergas.es. The HARMONY Alliance, via Dr Adrian Mosquera Orgueira, is responsible for evaluating and eventually accepting or refusing every request to disclose data and their related documents, in compliance with the ethical approval conditions, in compliance with applicable laws and regulations, and in conformance with the agreements in place with the involved subjects, the participating institutions, and all other parties directly or indirectly involved in the participation, conduct, development, management, and evaluation of this analysis.
