# Supplementary material for: Machine learning risk stratification strategy for multiple myeloma: Insights from the EMN–HARMONY Alliance platform
Source: Hemasphere. 2025 Oct 9;9(10):e70228. doi: 10.1002/hem3.70228 (PMC12509237; doi:10.1002/hem3.70228)
Supplement: Supplementary file 1 — Supporting Information. [file HEM3-9-e70228-s005.docx]

**Supplementary Data 1.** A comprehensive list of references for the trials used in the training and validation of the models.

1. Bringhen S, D'Agostino M, Paris L, et al. Lenalidomide-based induction and maintenance in elderly newly diagnosed multiple myeloma patients: updated results of the EMN01 randomized trial. Haematologica. 2020;105(7):1937-1947. doi:10.3324/haematol.2019.226407.
   - *EMN01 trial: Patients were enrolled across 58 Italian and 9 Czech centres between August 2009 and September 2012.*
2. Cavo M, Gay F, Beksac M, et al. Autologous haematopoietic stem-cell transplantation versus bortezomib-melphalan-prednisone, with or without bortezomib-lenalidomide-dexamethasone consolidation therapy, and lenalidomide maintenance for newly diagnosed multiple myeloma (EMN02/HO95): a multicentre, randomised, open-label, phase 3 study. Lancet Haematol. 2020;7(6):e456-e468. doi:10.1016/S2352-3026(20)30099-5
   - *EMN02 trial: Patients were enrolled between 25 February 2011 and 3 April 2014.*
3. Mateos MV, Oriol A, Martínez-López J, et al. Maintenance therapy with bortezomib plus thalidomide or bortezomib plus prednisone in elderly multiple myeloma patients included in the GEM2005MAS65 trial. Blood. 2012;120(13):2581-2588. doi:10.1182/blood-2012-05-427815
   - GEM2005MAS65 trial: The study was originally conducted in 63 Spanish centers between March 2006 and October 2008.
4. Lahuerta JJ, Paiva B, Vidriales MB, et al. Depth of Response in Multiple Myeloma: A Pooled Analysis of Three PETHEMA/GEM Clinical Trials. J Clin Oncol. 2017;35(25):2900-2910. doi:10.1200/JCO.2016.69.2517
   - *GEM2005MENOS65 trial: Patient enrollment commenced in March 2006. The actual primary completion date was December 2008, and the study was completed in December 2008.*
5. Mateos MV et al. Comparison of sequential vs alternating administration of bortezomib, melphalan and prednisone (VMP) and lenalidomide plus dexamethasone (Rd) in elderly patients with newly diagnosed multiple myeloma (MM): GEM2010MAS65 trial. Proc ASH 2013;Abstract 403.
   - *GEM2010MAS65 trial: Patient enrollment commenced in May 2010*.
6. Larocca A, Mina R, Offidani M, et al. First-line therapy with either bortezomib-melphalan-prednisone or lenalidomide-dexamethasone followed by lenalidomide for transplant-ineligible multiple myeloma patients: a pooled analysis of two randomized trials. Haematologica. 2020;105(4):1074-1080. doi:10.3324/haematol.2019.220657
   - *GIMEMA-MM-03-05 trial started in May 2006.*
7. Sonneveld P, Schmidt-Wolf IG, van der Holt B, et al. Bortezomib induction and maintenance treatment in patients with newly diagnosed multiple myeloma: results of the randomized phase III HOVON-65/ GMMG-HD4 trial [published correction appears in J Clin Oncol. 2012 Oct 10;30(29):3654]. J Clin Oncol. 2012;30(24):2946-2955. doi:10.1200/JCO.2011.39.6820
   - *HOVON-65 enrolled patients between May 2005 and May 2008.*
8. Zweegman S, van der Holt B, Mellqvist UH, et al. Melphalan, prednisone, and lenalidomide versus melphalan, prednisone, and thalidomide in untreated multiple myeloma. Blood. 2016;127(9):1109-1116. doi:10.1182/blood-2015-11-679415
   - *The HOVON87/NMSG18 trial recruited patients from March 12, 2009, to October 19, 2012.*
9. Mina R, Bonello F, Petrucci MT, et al. Carfilzomib, cyclophosphamide and dexamethasone for newly diagnosed, high-risk myeloma patients not eligible for transplant: a pooled analysis of two studies. Haematologica. 2021;106(4):1079-1085. Published 2021 Apr 1. doi:10.3324/haematol.2019.243428
   - *The IST-CAR-506 trial enrolled patients from June 21, 2011, to September 15, 2012, across 10 centers in Italy.*
10. Cavo M, Tacchetti P, Patriarca F, et al. Bortezomib with thalidomide plus dexamethasone compared with thalidomide plus dexamethasone as induction therapy before, and consolidation therapy after, double autologous stem-cell transplantation in newly diagnosed multiple myeloma: a randomised phase 3 study [published correction appears in Lancet. 2011 Nov 26;378(9806):1846]. Lancet. 2010;376(9758):2075-2085. doi:10.1016/S0140-6736(10)61424-9
    - *The MM-BO2005 trial enrolled patients from 73 sites in Italy between May 2006 and April 2008.*
11. Goldschmidt H, Mai EK, Dürig J, et al. Response-adapted lenalidomide maintenance in newly diagnosed myeloma: results from the phase III GMMG-MM5 trial. Leukemia. 2020;34(7):1853-1865. doi:10.1038/s41375-020-0724-1
    - *The MM5 trial recruited patients in Germany between July 2010 and October 2012.*
12. Larocca A, Bringhen S, Petrucci MT, et al. A phase 2 study of three low-dose intensity subcutaneous bortezomib regimens in elderly frail patients with untreated multiple myeloma. Leukemia. 2016;30(6):1320-1326. doi:10.1038/leu.2016.36
    - *The MMY2069 trial recruited subjects from October 2010 to August 2012.*
13. Jackson GH, Davies FE, Pawlyn C, et al. Lenalidomide maintenance versus observation for patients with newly diagnosed multiple myeloma (Myeloma XI): a multicentre, open-label, randomised, phase 3 trial. Lancet Oncol. 2019;20(1):57-73. doi:10.1016/S1470-2045(18)30687-9
    - *Between Jan 13, 2011, and Aug 11, 2017, 1917 patients were accrued to the maintenance treatment randomisation of the trial.*
14. Bonello F, Pulini S, Ballanti S, et al. Lenalidomide Maintenance with or without Prednisone in Newly Diagnosed Myeloma Patients: A Pooled Analysis. Cancers (Basel). 2019;11(11):1735. Published 2019 Nov 5. doi:10.3390/cancers11111735
    - *The RV-MM-EMN-441 study began in July 2009.*
15. Palumbo A, Gay F, Falco P, et al: Bortezomib as induction before autologous transplantation, followed by lenalidomide as consolidation-maintenance in untreated multiple myeloma patients. J Clin Oncol 28:800-807, 2010
    - *The RV-MM-PI-114 trial enrolled 102 patients from 17 Italian centers between October 2005 and July 2007.*
16. Palumbo A, Cavallo F, Gay F, et al. Autologous transplantation and maintenance therapy in multiple myeloma. N Engl J Med. 2014;371(10):895-905. doi:10.1056/NEJMoa1402888
    - *The RV-MM-PI-209 trial recruited patients from November 2007 to July 2009 at 62 centers in Italy and Israel.*
17. San Miguel JF, Schlag R, Khuageva NK, et al. Bortezomib plus melphalan and prednisone for initial treatment of multiple myeloma. N Engl J Med. 2008;359(9):906-917. doi:10.1056/NEJMoa0801479
    - *The VISTA trial recruited patients from December 2004 to September 2006.*
18. Dimopoulos MA, Oriol A, Nahi H, et al. Daratumumab, Lenalidomide, and Dexamethasone for Multiple Myeloma. N Engl J Med. 2016;375(14):1319-1331. doi:10.1056/NEJMoa1607751
    - *The POLLUX trial randomized patients between June 16, 2014, and July 14, 2015, at 135 sites across 18 countries in North America, Europe, and the Asia Pacific region.*

**Supplementary Data 2.** Computational environment and software used for this study.

All analyses were performed in **R 3.6.3 (2020‑02‑29)** on a **CentOS 7 (x86_64‑pc‑linux‑gnu, 64‑bit)** platform. Core packages were *randomForestSRC 2.10.1* for model training, *survival 3.5‑5* and *timeROC 0.4* for time‑to‑event inference, *survminer 0.4.9, riskRegression_2020.12.08 knitr_1.31* for cumulative risk analysis, *ggplot2 3.5.1* for visualisation, and *SparkR 2.2.0* to interface with an Apache Spark 2.2 cluster for distributed preprocessing. Auxiliary libraries (e.g., *dplyr 1.0.5*, *tidyr 1.1.3*, *caret 6.0‑86*) supported data wrangling and resampling.

Models were trained on an on‑premises HPC node equipped with **2 × Intel Xeon E5‑2680 v4 (32 logical cores) and 256 GB RAM**; no GPU acceleration was required. Analyses were executed via fully scripted pipelines. A random seed was fixed (set.seed(1234)) for all bootstrap resampling and **out‑of‑bag (OOB) error estimation** inherent to the random‑forest algorithm.
