# Supplementary figures and images for: Machine learning risk stratification strategy for multiple myeloma: Insights from the EMN–HARMONY Alliance platform
Source: Hemasphere. 2025 Oct 9;9(10):e70228. doi: 10.1002/hem3.70228 (PMC12509237; doi:10.1002/hem3.70228)

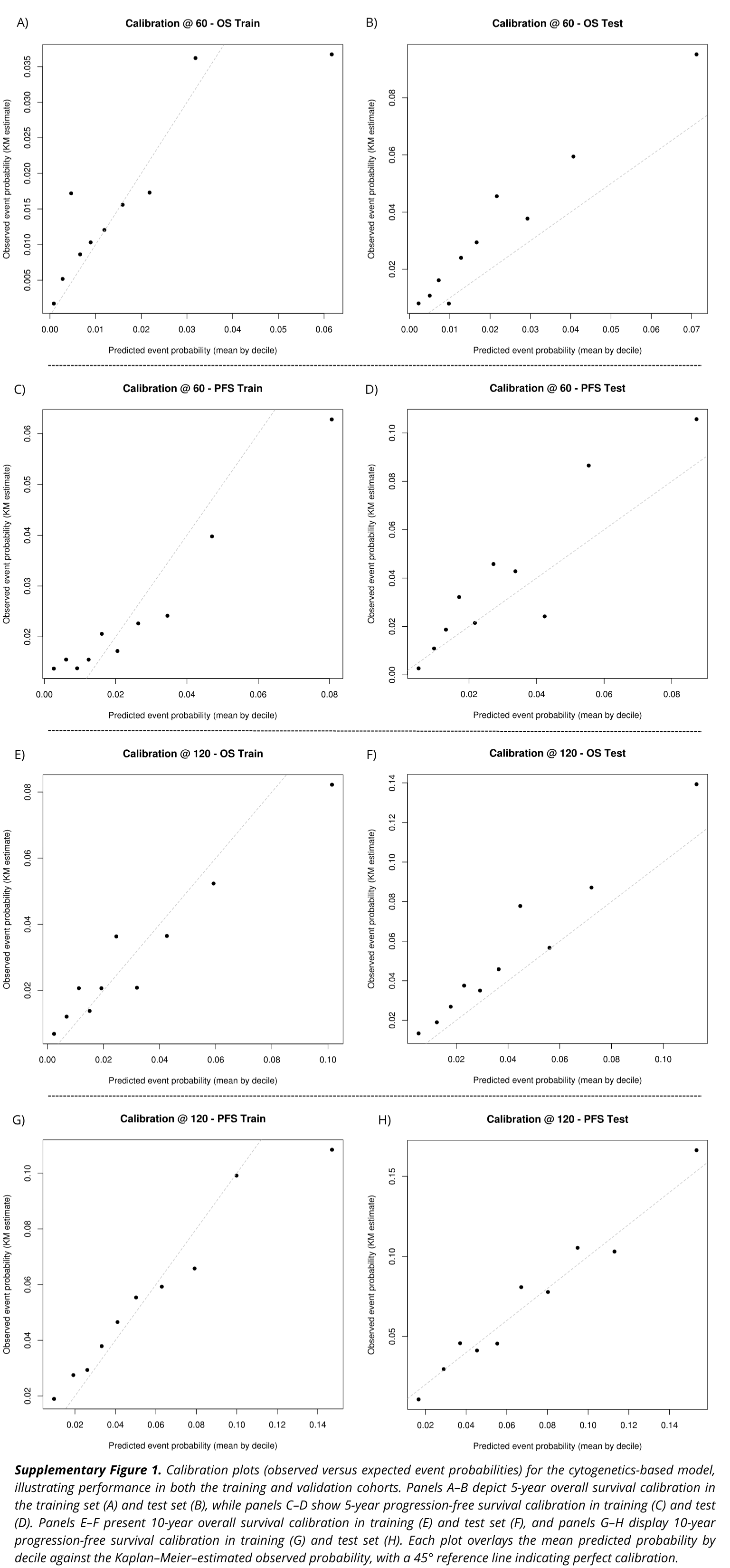

Supplement: Supplementary file 2 — Supporting Information. [file HEM3-9-e70228-s006.png]

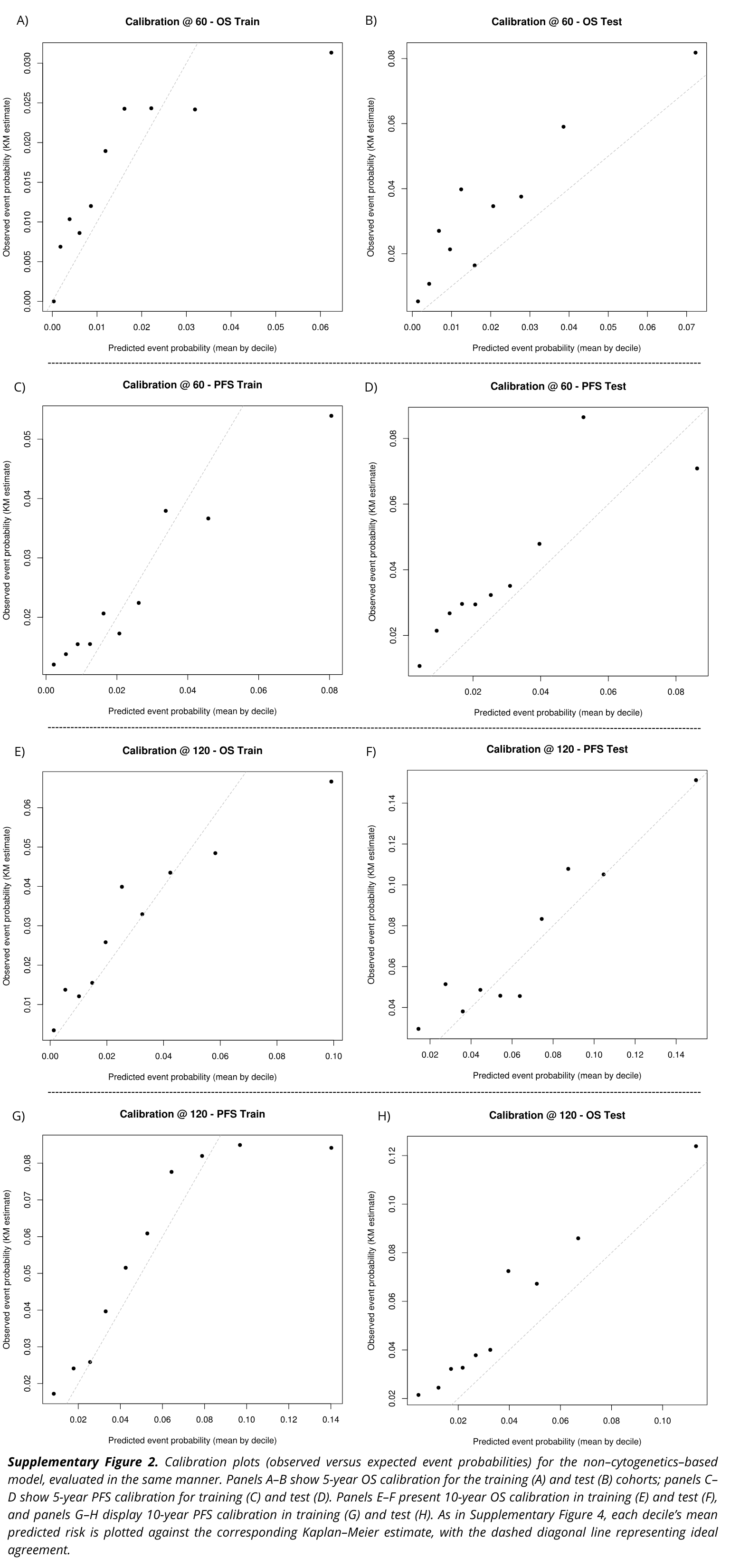

Supplement: Supplementary file 3 — Supporting Information. [file HEM3-9-e70228-s003.png]

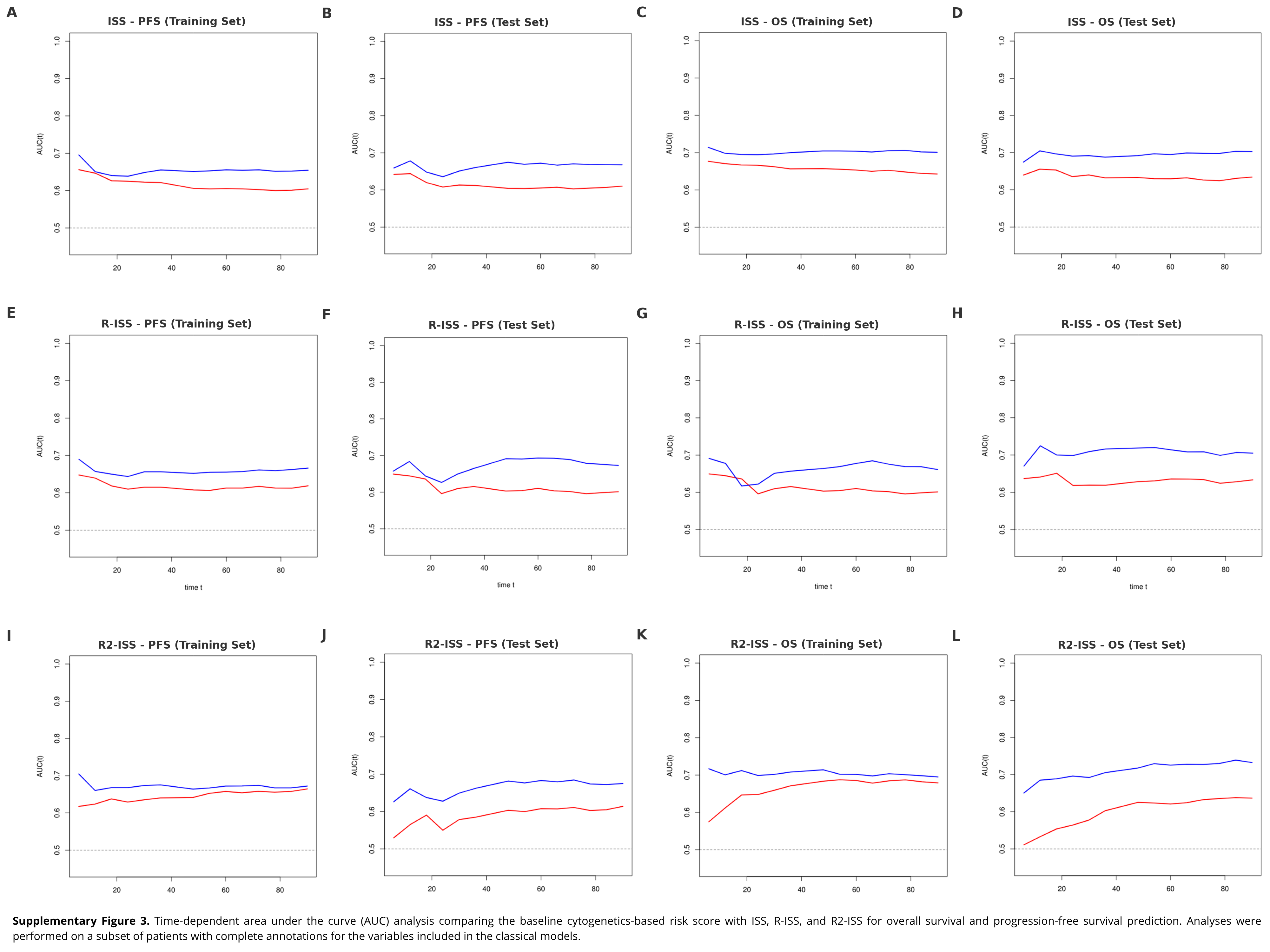

Supplement: Supplementary file 4 — Supporting Information. [file HEM3-9-e70228-s004.png]

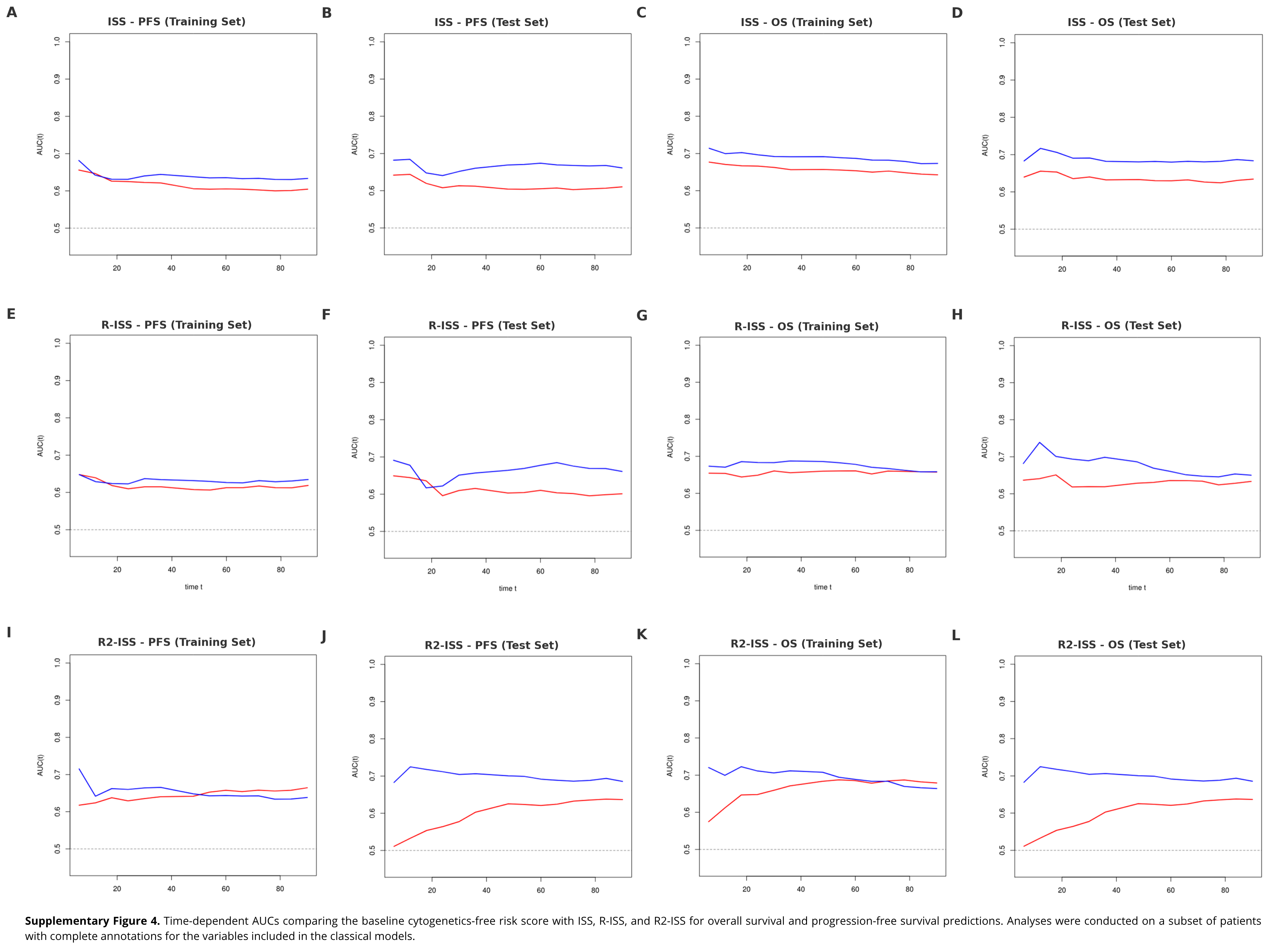

Supplement: Supplementary file 5 — Supporting Information. [file HEM3-9-e70228-s008.png]

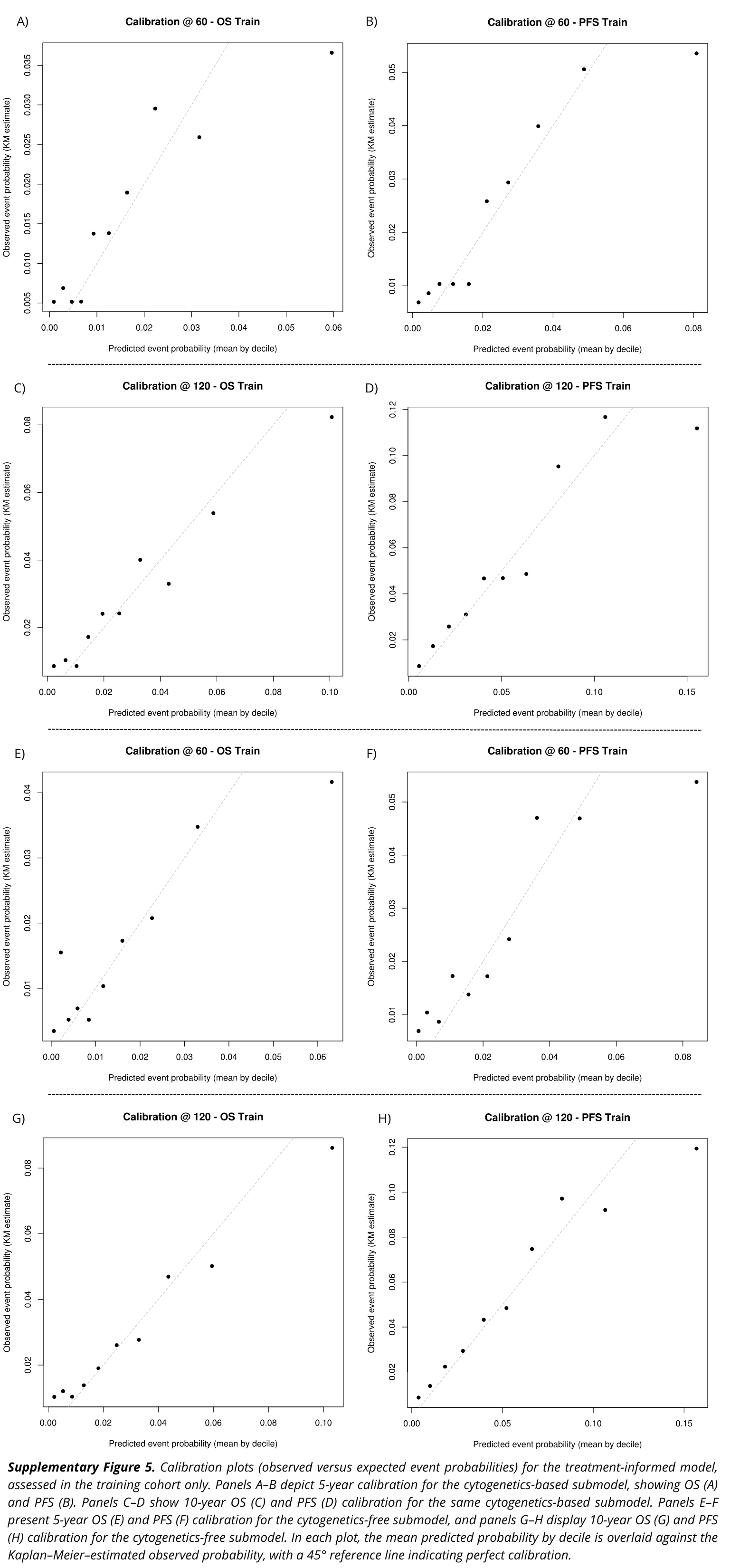

Supplement: Supplementary file 6 — Supporting Information. [file HEM3-9-e70228-s010.png]

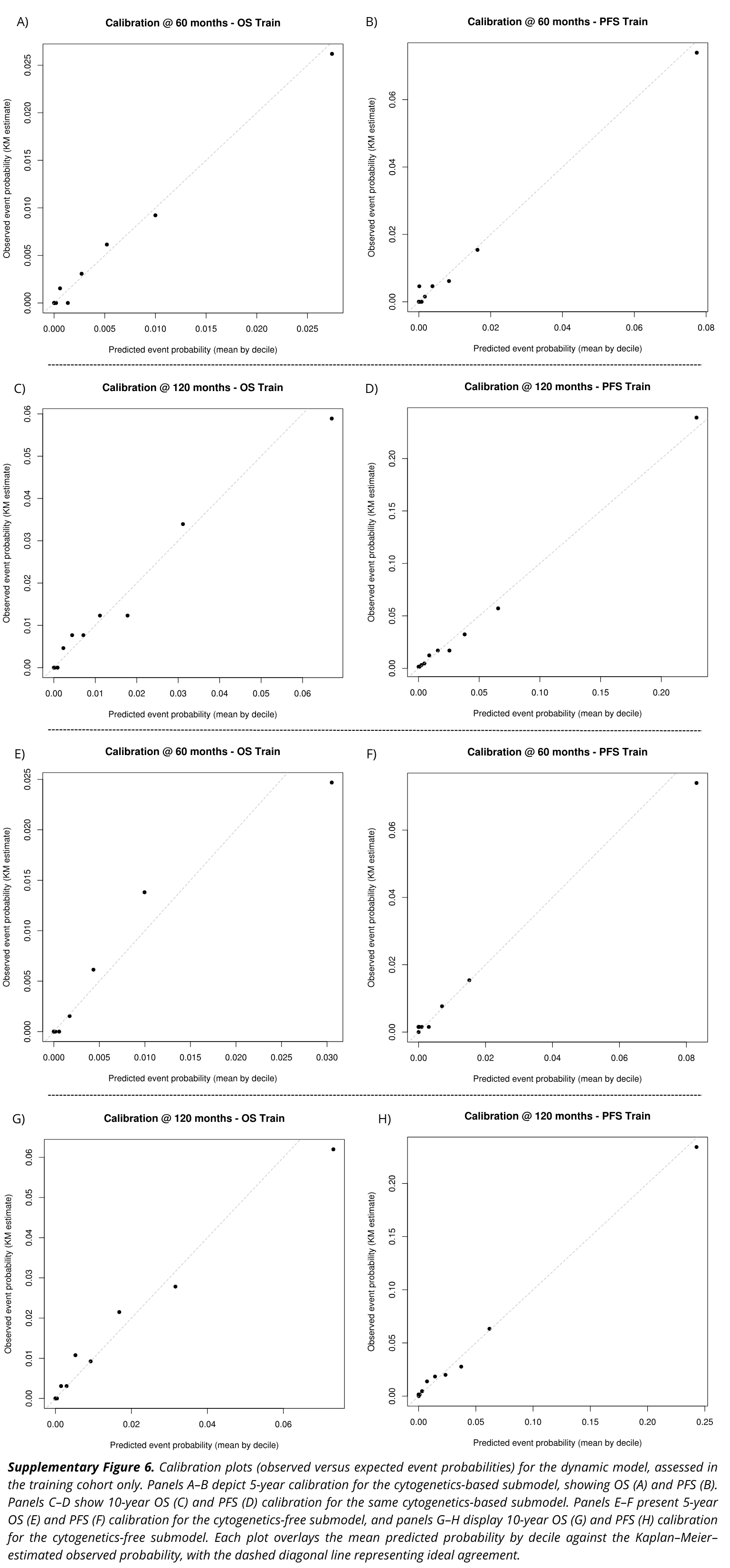

Supplement: Supplementary file 7 — Supporting Information. [file HEM3-9-e70228-s002.png]

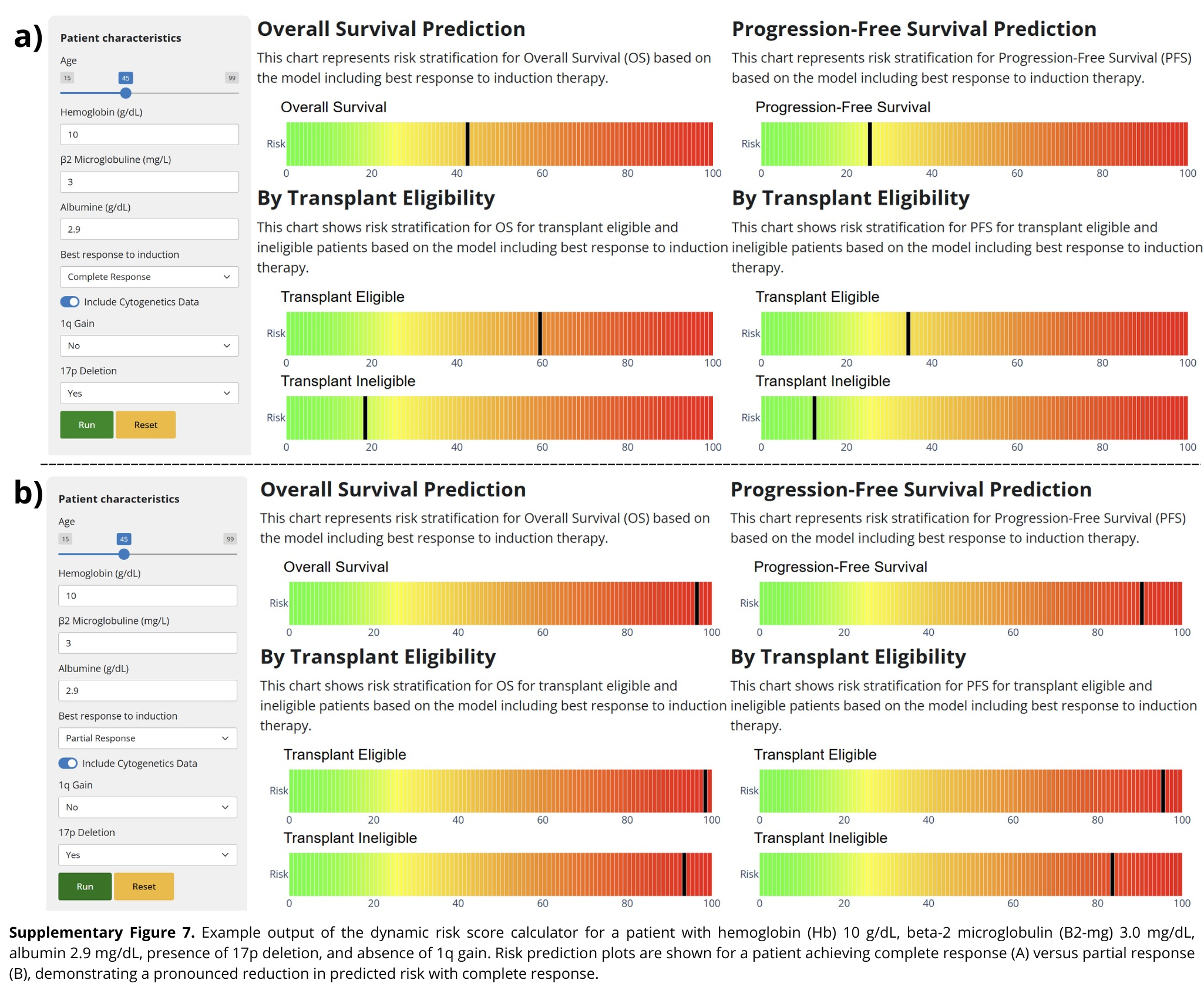

Supplement: Supplementary file 8 — Supporting Information. [file HEM3-9-e70228-s001.png]
