# Supplementary material for: Machine learning risk stratification strategy for multiple myeloma: Insights from the EMN–HARMONY Alliance platform
Source: Hemasphere. 2025 Oct 9;9(10):e70228. doi: 10.1002/hem3.70228 (PMC12509237; doi:10.1002/hem3.70228)
Supplement: Supplementary file 9 — Supporting Information. [file HEM3-9-e70228-s009.docx]

| **Supplementary Table 1**. C‑index values and their 95 % confidence intervals for predicting progression‑free survival (PFS) and overall survival (OS) in the training and test datasets, stratified by the presence of > 50 % missing data, for both cytogenetics‑based and cytogenetics‑free models. | | | | |
| --- | --- | --- | --- | --- |
| **Model** | **Missing Data** | **Endpoint** | **Training** | **Test** |
| Cytogenetics-Based Model (6 variables) | ≤50% missing | OS | 0.660 (0.650–0.671) | 0.668 (0.653–0.683) |
|  |  | PFS | 0.607 (0.598–0.616) | 0.617 (0.605–0.630) |
|  | >50% missing | OS | 0.610 (0.529–0.690) | 0.640 (0.610–0.671) |
|  |  | PFS | 0.558 (0.487–0.628) | 0.584 (0.560–.609) |
| Cytogenetics-Free Model (4 variables) | ≤50% missing | OS | 0.660 (0.649–0.670) | 0.666 (0.651–0.681) |
|  |  | PFS | 0.605 (0.596–0.614) | 0.619 (0.607–0.632) |
|  | >50% missing | OS | 0.608 (0.525–0.691) | 0.636 (0.605–0.667) |
|  |  | PFS | 0.571 (0.497–0.645) | 0.598 (0.574–0.623) |
