# Supplementary material for: Machine learning risk stratification strategy for multiple myeloma: Insights from the EMN–HARMONY Alliance platform
Source: Hemasphere. 2025 Oct 9;9(10):e70228. doi: 10.1002/hem3.70228 (PMC12509237; doi:10.1002/hem3.70228)
Supplement: Supplementary file 10 — Supporting Information. [file HEM3-9-e70228-s007.docx]

| **Supplementary Table 2.** AIC values derived from Fine-Gray regression analyses using progression as the  event of interest and death without prior progression as a competing risk. Results are shown for the ISS, R-ISS and R2-ISS in relation to risk scores generated by random forest models for overall survival and progression-free survival, considering both cytogenetics-based and cytogenetics-free approaches. Analyses were performed in patient subsets without missing data necessary for calculating ISS, R-ISS, or R2-ISS (as applicable), in order to minimize comparative bias. | | | | | |
| --- | --- | --- | --- | --- | --- |
| **Cytogenetics-based score** | | | | | |
| **Clinical score** | **Data set** | **Outcome** | **AIC (classical)** | **AIC (ML)** | **ΔAIC** |
| **ISS** | Train | OS | 85 580 | 85 368 | -212 |
|  | Test | OS | 40 040 | 39 912 | -128 |
|  | Train | PFS | 85 580 | 85 314 | -266 |
|  | Test | PFS | 40 040 | 39 936 | -104 |
| **RISS** | Train | OS | 45 680 | 45 594 | -86 |
|  | Test | OS | 17 058 | 16 980 | -78 |
|  | Train | PFS | 45 680 | 45 564 | -116 |
|  | Test | PFS | 17 058 | 16 990 | -68 |
| **R‑2 ISS** | Train | OS | 23 558 | 23 558 | 0 |
|  | Test | OS | 12 950 | 12 864 | -86 |
|  | Train | PFS | 23 558 | 23 532 | -26 |
|  | Test | PFS | 12 950 | 12 886 | -64 |
| **Cytogenetics-free score** | | | | | |
| **Clinical score** | **Data set** | **Outcome** | **AIC (classical)** | **AIC (ML)** | **ΔAIC** |
| **ISS** | Train | OS | 85 580 | 85 480 | -100 |
|  | Test | OS | 40 040 | 39 956 | -84 |
|  | Train | PFS | 85 580 | 85 472 | -108 |
|  | Test | PFS | 40 040 | 39 948 | -92 |
| **RISS** | Train | OS | 45 680 | 45 656 | -24 |
|  | Test | OS | 17 058 | 17 014 | -44 |
|  | Train | PFS | 45 680 | 45 680 | 0 |
|  | Test | PFS | 17 058 | 17 014 | -44 |
| **R‑2 ISS** | Train | OS | 23 558 | 23 576 | 18 |
|  | Test | OS | 12 950 | 12 896 | -54 |
|  | Train | PFS | 23 558 | 23 582 | 24 |
|  | Test | PFS | 12 950 | 12 896 | -54 |
